# Extracellular Vesicles of *Streptococcus anginosus* Mediate Gastritis via Epithelial Barrier Disruption and Macrophage‐driven Inflammation

**DOI:** 10.1002/advs.202512494

**Published:** 2026-01-30

**Authors:** Ying Gong, Lina Duan, Jie Xiao, Yulu Deng, Hongxia Wang, Yajie Zhang, Xiumei Hu, Haifang Wang, Taixue An, Xin Li, Yurong Qiu, Lei Zheng, Haixia Li

**Affiliations:** ^1^ Department of Laboratory Medicine Nanfang Hospital Southern Medical University Guangzhou P. R. China; ^2^ Guangdong Engineering and Technology Research Center for Rapid Diagnostic Biosensors; Guangdong Provincial Key Laboratory of Precision Medical Diagnostics; Guangdong Provincial Key Laboratory of Single‐cell and Extracellular Vesicles Guangdong Provincial Clinical Research Center for Laboratory Medicine Guangzhou Guangdong P. R. China; ^3^ Department of Internal Medicine Division of Hematology University of Maastricht Maastricht Netherlands; ^4^ State Key Laboratory of Multi‐organ Injury Prevention and Treatment Nanfang Hospital Southern Medical University Guangzhou P. R. China

**Keywords:** aspartate, epithelial barrier dysfunction, extracellular vesicles (EVs), gastritis pathogenesis, non‐H. pylori gastritis, pro‐inflammatory cytokines, streptococcus anginosus

## Abstract

Recent studies suggest *Streptococcus anginosus* (*SA*) contributes to gastric disease beyond Helicobacter pylori, yet its pathogenic mechanisms remain unclear. This study demonstrates that *SA*‐derived extracellular vesicles (*SA*‐EVs) accumulate in gastric tissue, enter epithelial cells, and induce acute gastritis characterized by neutrophil infiltration and elevated cytokines (TNF‐α, IL‐6, IL‐17A). Chronic exposure leads to sustained inflammation, tight junction disruption (Claudin‐18, Occludin, ZO‐1), and mucosal damage. Proteomic analysis identified TMPC and FBP62 as virulence‐associated proteins enriched in *SA*‐EVs, while transcriptomics revealed activation of macrophage polarization and cytokine‐receptor pathways. Metabolomic profiling indicated dysregulated aspartate metabolism and inflammation‐related metabolic changes, alongside increased gut *SA* abundance. Notably, genetic deletion of *Tmpc* or *Fbp62* significantly attenuated *SA*‐EVs pathogenicity in vivo, reducing gastric inflammation, cytokine production, and macrophage infiltration. These findings establish *SA*‐EVs as key mediators of non‐*H. pylori* gastritis, with TMPC and FBP62 orchestrating epithelial barrier disruption, immune activation, and metabolic dysregulation, highlights their potential as therapeutic targets.

## Graphical abstract

*Streptococcus anginosus* extracellular vesicles (*SA*‐EVs) accumulate in gastric tissue, disrupt epithelial tight junctions, and induce gastritis characterized by neutrophil infiltration and elevated cytokines (TNF‐α, IL‐6, IL‐17A). Proteomics identifies TMPC and FBP62 as key *SA*‐EVs virulence factors; their genetic deletion attenuates inflammation. Transcriptomics reveals macrophage polarization and Th17 pathway activation, while fecal metabolomics shows aspartate enrichment and gut microbiota dysbiosis. This study establishes *SA*‐EVs as central mediators of non‐*H. pylori* gastritis, highlighting them as potential diagnostic and therapeutic targets. Created in BioRender. Li, B. (2026) https://BioRender.com/9yyi3ro.

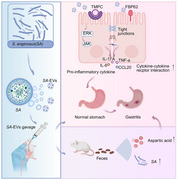

## 1 Introduction

Gastritis, characterized by inflammation of the gastric mucosa, is a common condition with diverse causes, including microbial infections, nonsteroidal anti‐inflammatory drugs (NSAIDs), excessive alcohol consumption, autoimmune diseases, and stress [1]. Helicobacter pylori (*H. pylori*) is a Gram‐negative, transmissible pathogen, with 1%–10% of treatment individuals developing clinical complications such as peptic ulcers, gastric atrophy, intestinal metaplasia, and, in some cases, progressing to gastric cancer or mucosa‐associated lymphoid tissue (MALT) lymphoma [2]. *H. pylori* primarily exerts its effects by modulating host immunity, particularly through altering the functions of innate and adaptive immune cells, thereby suppressing inflammatory responses that would otherwise hinder its survival. This immune evasion enables the bacterium to persist in the gastric mucosa, ultimately leading to gastritis [3]. While *H. pylori* is the most well‐documented microbial agent associated with gastritis, peptic ulcers, and gastric cancer [4], recent evidence suggests that other bacteria may also contribute to gastric inflammation, including *Prevotella stomatis, Dialister pneumosintes, Solobacterium exigua, Parvimonas micra*, and *Streptococcus anginosus* [5, 6, 7, 8]. These findings underscore a broader and underexplored spectrum of microbial involvement in gastritis pathogenesis.

Among these potential contributors, *SA*, a member of the viridans group streptococci, has garnered attention due to its emerging association with gastric pathologies [9]. While typically regarded as a commensal organism of the oral cavity, gastrointestinal tract, and upper respiratory tract, *SA* is capable of causing invasive infections such as abscesses [10] and infective endocarditis [11]. In previous studies, *SA* has been shown to exhibit pathogenic potential by binding to various host proteins, including adhesins, hyaluronic acid receptors, collagen, and others [12]. What's more, another study revealed that SanA nuclease should be recognized as a key virulence factor of *SA*, contributing to the severity of monospecies purulent infections [9]. Furthermore, Fu et al. identify *SA* as a pathogen that promotes gastric tumorigenesis through direct interactions with gastric epithelial cells, mediated by the TMPC (membrane‐associated lipoprotein of *Treponema pallidum* (*T. pallidum*)) ‐ANXA2 (Annexin A2) ‐MAPK (mitogen‐activated protein kinases) axis [5]. Moreover, Kodama et al. also demonstrated that fibronectin‐binding protein homologue FBP62 of *SA* is a potent virulence factor [13]. However, the detailed mechanisms underlying *SA*‐mediated gastric disease remain insufficiently defined.

Recent microbiological studies have highlighted the pivotal role of bacterial extracellular vesicles (BEVs) secreted by intestinal microbes—including commensals, probiotics, and pathogens—as key mediators that profoundly influence the intestinal microenvironment and human health [14, 15, 16, 17]. In addition, the use of BEVs is considered a promising therapeutic strategy for complex diseases by modulating the diversity and abundance of intestinal microbiota and preserving intestinal homeostasis [18, 19]. BEVs are lipid bilayer‐enclosed structures, typically ranging from 20 to 500 nm in diameter, and are secreted by bacteria during growth [16, 20]. Similar to extracellular vesicles secreted by eukaryotic cells, BEVs carry a diverse cargo of proteins, enzymes, DNA, RNA, peptidoglycans, and lipids, facilitating both intra‐ and inter‐kingdom communication [21, 22, 23, 24]. Growing evidence indicates that BEVs can be generated through multiple biogenetic pathways, resulting in distinct subtypes with diverse molecular cargo and potentially different biological functions [25]. For example, EVs from *H. pylori contain* virulence factors such as CagA and VacA, which modulate host cell signaling and provoke inflammation [26]. *Fusobacterium nucleatum* can deliver FadA molecules through vesicles, exacerbating rheumatoid arthritis [27]. Whether *SA*‐EVs exert similar effects on the gastric mucosa is unknown, presenting a critical gap in understanding their role in gastritis pathogenesis. Its potential role in gastritis remains underexplored, particularly concerning its ability to release EVs, and whether these vesicles have a pathogenic effect.

In this study, we found that *SA*‐EVs can carry relevant virulence factors, such as TMPC and FBP62, which collectively contribute to the induction of both acute and chronic gastritis. We demonstrate that *SA*‐EVs disrupt epithelial barrier integrity by downregulating tight junction proteins, and elicit robust inflammatory responses as evidenced by upregulation of cytokine signaling pathways and macrophage‐associated gene expression. Furthermore, fecal metabolomic profiling revealed a marked increase in aspartate and related metabolic pathways, suggesting that *SA*‐EVs‐induced metabolic alterations may further amplify mucosal inflammation. These findings provide novel insights into the pathogenic mechanisms of *SA*‐EVs and their critical role in non‐*H. pylori*‐associated gastritis.

## 2 Results

### 2.1 SA‐EVs Accumulate in the Gastric Mucosa and are Internalized by Epithelial Cells

Previous studies have demonstrated that *SA* can colonize the gastric mucosa and interact with gastric epithelial cells via TMPC receptors, thereby promoting the onset and progression of gastritis [5]. Under normal physiological conditions, the mucosal immune barrier serves as a critical defense mechanism, limiting *SA* access to the blood [28]. Interestingly, *F. Necleatum* has been shown to exacerbate rheumatoid arthritis by releasing FadA‐containing extracellular vesicles (EVs) [27], highlighting the significant role of EVs as mediators between the gut microbiome and host immune system [29, 30]. However, whether *SA* transmits virulence determinants through extracellular vesicles remains to be further explored.

**FIGURE 1:**
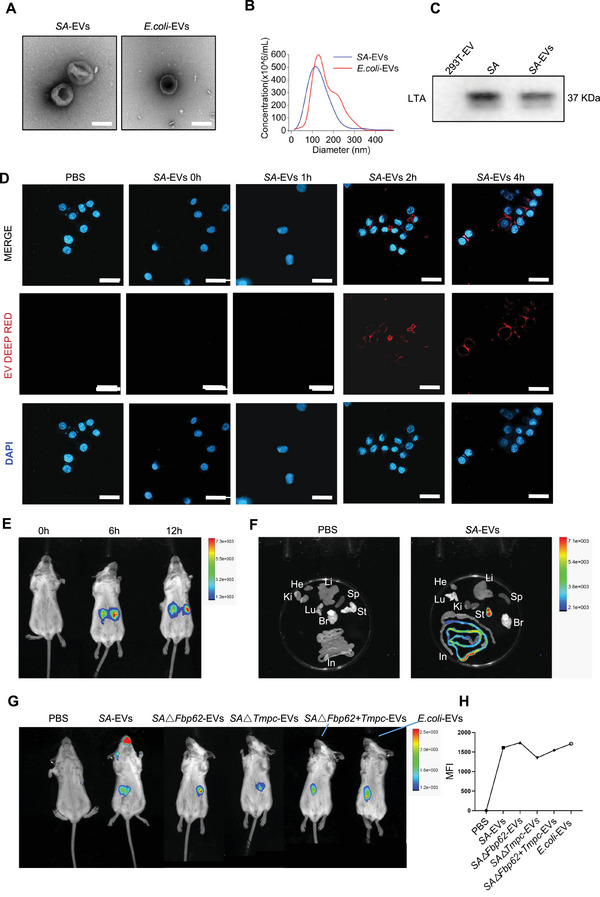
Characterization and gastric uptake of *SA*‐EVs. (A) TEM images of *SA*‐EVs and *E.coli*‐EVs with a scale bar of 100 nm. (B) Size distribution analysis of *SA*‐EVs and *E.coli*‐EVs performed using NTA. (C) Lipoteichoic acid (LTA) expression in Western blot images of *SA*‐EVs. (D) The intake assay of Deep red labelled *SA*‐EVs on gastric mucosa epithelium (GES‐1) cells. Scale bars=100 µm. (E) Fluorescence imaging of whole‐body uptakewas performed 0, 6, 12 h after intragastric administration of *SA*‐EVs. (F) Fluorescence imaging of dissected organs from the same mice, showing the distribution of *SA*‐EVs across different tissues. Fluorescence signals indicate the specific uptake and localization of *SA*‐EVs compared to the control group. (G) Fluorescence imaging of systemic uptake was performed 24 h after intragastric administration of *SA*‐EVs, PBS, *SA*△*Fbp62*‐EVs, *SA*△*Tmpc*‐EVs, *SA*△*Fbp6*2+*Tmpc*‐EVs, and *E.coli*‐EVs, and the fluorescence intensity was statistically analyzed. He: heart, Lu: lung, Ki: kidney, Li: liver, Sp: spleen, St: stomach, Br: brain, In: intestine.

To investigate the role of *SA*‐EVs in gastritis, we cultured *SA* and purified its EVs, using *Escherichia coli*‐derived EVs (*E.coli*‐EVs) as a control. (Figure 1A). The isolated *SA*‐EVs and *E. coli*‐EVs were approximately 100 nm in diameter (Figure 1B) and enriched in lipoteichoic acid (LTA) molecules (Figure 1C). *SA*‐EVs were inoculated onto MH agar plates, and no viable *SA* was detected. This proved the purity of the product (Figure S7A). Cellular uptake experiments confirmed that over time, within 2 h, *SA*‐EVs are internalized by gastric epithelial cells (Figure 1D). Furthermore, fluorescence imaging in a mouse model revealed that intragastrically administered *SA*‐EVs in gastric tissues, accumulated in the gastric for over 24 h (Figure 1E–I). These findings provide compelling evidence that *SA*‐EVs can specifically target and persist within gastric tissue, establishing a foundation for further studies into their pathogenic role in gastritis.

### 2.2 Acute Gastritis and Pro‐Inflammatory Cytokine Induction After Short‐Term SA‐EVs Exposure

**FIGURE 2:**
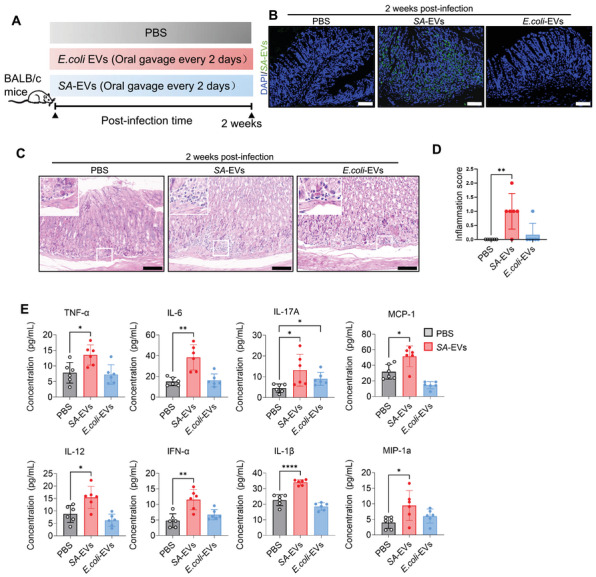
*SA*‐EVs colonize the gastric mucosa and induce acute gastric inflammation. (A) BALB/c female mice (*n* = 6 per group) were orally gavaged with *SA*‐EVs, PBS, *E.coli*‐EVs for two weeks. (B) Representative fluorescence in situ hybridization (FISH) images of gastric tissue sections from mice gavaged with *SA*‐EVs, PBS, *E.coli*‐EVs at two weeks post‐infection. (Blue indicates nuclei; green indicates *SA* probe). Scale bars represent 50 µm. (C) Representative hematoxylin and eosin (H&E) staining images of stomach tissue from mice treated *wit*h *SA*‐EVs, PBS, *E.coli*‐EVs at two weeks post‐infection. Scale bars = 50 µm. (D) Gastric inflammation scores for the different groups: score 0, no inflammation; score 1, mild inflammation; score 2, moderate inflammation; score 3, severe inflammation; score 4, critical inflammation. (E) Serum levels of TNF‐α, IL‐6, IL‐17, MIP‐1a, IL‐12, IFN‐a, IL‐1β, and MCP‐1 were upregulated following *SA*‐EVs infection at two weeks post‐infection. Data is shown as mean ± SD, with dots representing individual donors (average of technical duplicates, n=6). Statistical differences between groups were determined using one‐way ANOVA with Tukey post‐tests. *p* < 0.05 (*); *p* < 0.01 (**); *p* < 0.001 (***); *p* < 0.0001 (****); ns: not significant.

To evaluate the pathogenic potential of *SA*‐EVs, mice were gavaged with *SA*‐EVs (20 µg) every two days over a two‐week period (Figure 2A). Meanwhile, mice treated with PBS and *E. coli*‐EVs (20 µg) served as controls. Two weeks post‐infection, fluorescence in situ hybridization (FISH) detected DNA from *SA* in the basal region of the gastric mucosa of mice in the *SA*‐EVs group (Figure 2B), providing direct evidence of their ability to localize to specific regions of the gastric tissue in vivo. It has been reported that BEVs can carry bacterial DNA, and extracellular vesicle DNA (EV‐DNA) holds potential as a biomarker for disease diagnosis [31]. Therefore, we used a FISH probe to localize *SA*‐EVs. Histological analysis revealed acute inflammation in *SA*‐EVs‐treated mice, characterized by significant inflammatory cells infiltration, as observed in H&E‐stained sections (Figure 2C). According to an established histological scoring scheme, 16% (1 out of 6) of *SA*‐EVs‐treated mice exhibited mild to moderate inflammation, while 67% (4 out of 6) displayed mild inflammation (Figure 2D). Notably, levels of pro‐inflammatory cytokines, including TNF‐α, IL‐6, IL‐17A, MCP‐1, IL‐12, IFN‐a, IL‐1β, and MIP‐1a, were significantly elevated in *SA*‐EVs‐treated mice compared to controls (Figure 2E). The above experimental results are similar to those found by Fu et al. that *SA* causes gastritis. These findings demonstrate that *SA*‐EVs can induce acute gastric inflammation in mice and highlight the associated upregulation of pro‐inflammatory chemokines as a critical molecular mechanism. This study provides robust experimental evidence for the role of *SA*‐EVs in the pathogenesis of gastric disease.

### 2.3 Chronic SA‐EVs Exposure Causes Sustained Gastritis With Epithelial Barrier Disruption

**FIGURE 3:**
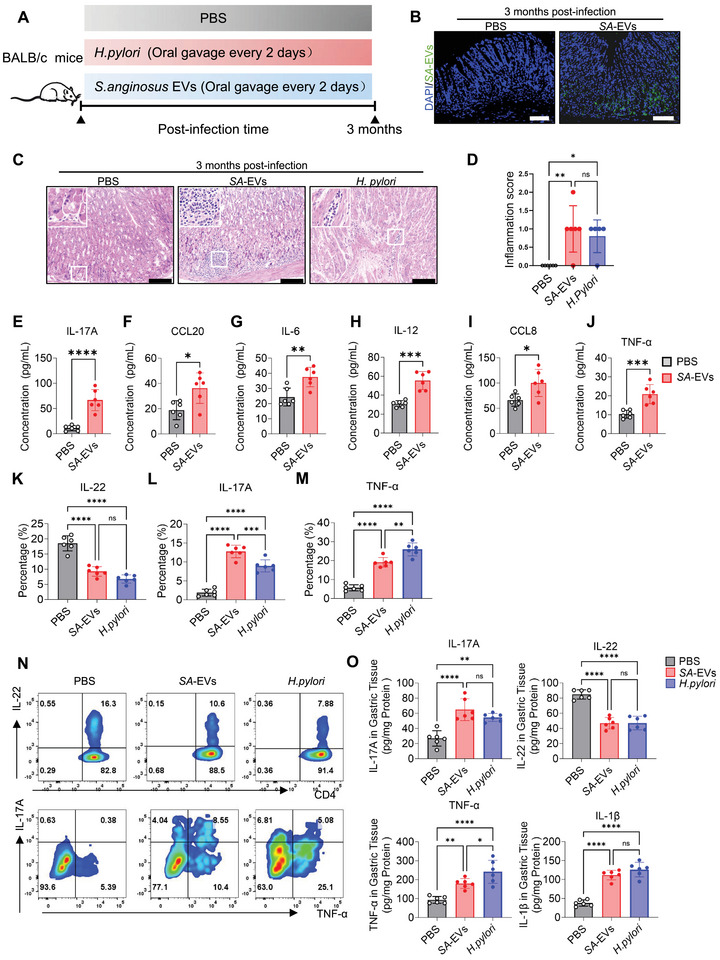
Chronic exposure to *SA*‐EVs induces sustained gastric inflammation. (A) BALB/c female mice (n=6 per group) were orally gavaged with either *SA*‐EVs, *H. pylori* or PBS for two months. (B) Representative fluorescence in situ hybridization (FISH) images of gastric tissue sections from mice gavaged with *SA*‐EVs or PBS at two months post‐infection. (Blue indicates nuclei; green indicates *S. anginosus* probe). Scale bars represent 50 µm. (C) Representative hematoxylin and eosin (H&E) staining images of stomach tissue from mice treated with *SA*‐EVs, *H. pylori* or PBS at two months post‐infection. Scale bars=50 µm. (D) Gastric inflammation scores for the different groups: score 0, no inflammation; score 1, mild inflammation; score 2, moderate inflammation; score 3, severe inflammation; score 4, critical inflammation. (E‐M) Serum levels of IL‐17A, CCL20, IL‐6, IL‐12, CCL8, and TNF‐a, were upregulated following *SA*‐EVs infection at two weeks post‐infection. (L–O) Flow pattern(O) and statistical analysis(L‐N) of IL‐22, IL‐17A, and TNF‐α in gastric tissue following 3 months of intragastric administration. (P) ELISA analysis of TNF‐α, IL‐17a, CCL20, and IL‐1β in gastric tissue following 3 months of intragastric administration. Data is shown as mean ± SD, with dots representing individual donors (average of technical duplicates, *n* = 6). Statistical differences between groups were determined using one‐way ANOVA with Tukey post‐tests. *p* < 0.05 (*); *p* < 0.01 (**); *p* < 0.001 (***); *p* < 0.0001 (****); ns: not significant.

To investigate the long‐term effects of *SA‐*EVs infection on the stomach, we developed a chronic infection model. Mice were administered *SA*‐EVs via gavage every two days over a three‐month period (Figure 3A), with PBS‐treated mice serving as negative controls and *H. pylori* ‐treated mice as positive controls. *SA*‐EVs infection had no significant effect on body weight (Figure S3A), or food intake (Figure S2B). *SA*‐EVs did not induce inflammation in the colon (Figure S3C,D). Furthermore, liver function markers, including alanine transaminase (ALT), aspartate aminotransferase (AST), albumin (ALB), and or liver‐to‐body weight ratio, remained within normal ranges in *SA*‐EVs‐treated mice (Figure S3E,F). *SA*‐EVs infection in the stomach was confirmed through PCR analysis of bacterial DNA and inoculation of gastric tissues onto MH plates (Figure S3G–J). FISH confirmed the consistent localization of *SA*‐EVs in the gastric mucosa throughout the experimental period (Figure 3B).

Chronic inflammation, characterized by persistent neutrophil infiltration and lymphocyte aggregation, was observed in mice treated with *SA*‐EVs and *H. pylori* (Figure 3C). Notably, *SA*‐EVs‐treated mice exhibited significantly elevated gastric inflammation scores compared to PBS controls (Figure 3D). What's more, levels of pro‐inflammatory cytokines, including IL‐17A, CCL20, IL‐6, IL‐12, CCL8, and TNF‐a, were significantly elevated in *SA*‐EVs‐treated mice compared to controls (Figure 3E–J). In addition, flow cytometric analysis of gastric tissues from mice after three months of *SA*‐EVs administration revealed significant changes in inflammatory factors. Specifically, IL‐17A and TNF‐α were significantly elevated in both the *SA*‐EVs and *H. pylori* groups compared to the PBS group (Figure 3K–N). Furthermore, ELISA analysis of stomach tissue confirmed the alterations in the aforementioned inflammatory factors (Figure 3O and Figure S1A). These findings underscore the sustained inflammatory effects of *SA*‐EVs on gastric tissue and provide valuable experimental evidence for elucidating the mechanisms underlying chronic gastric diseases associated with *SA*‐EVs infection.

### 2.4 SA‐EVs Disrupt Gastric Barrier Function and Alter Gastric Microbiota Homeostasis in Mice

**FIGURE 4:**
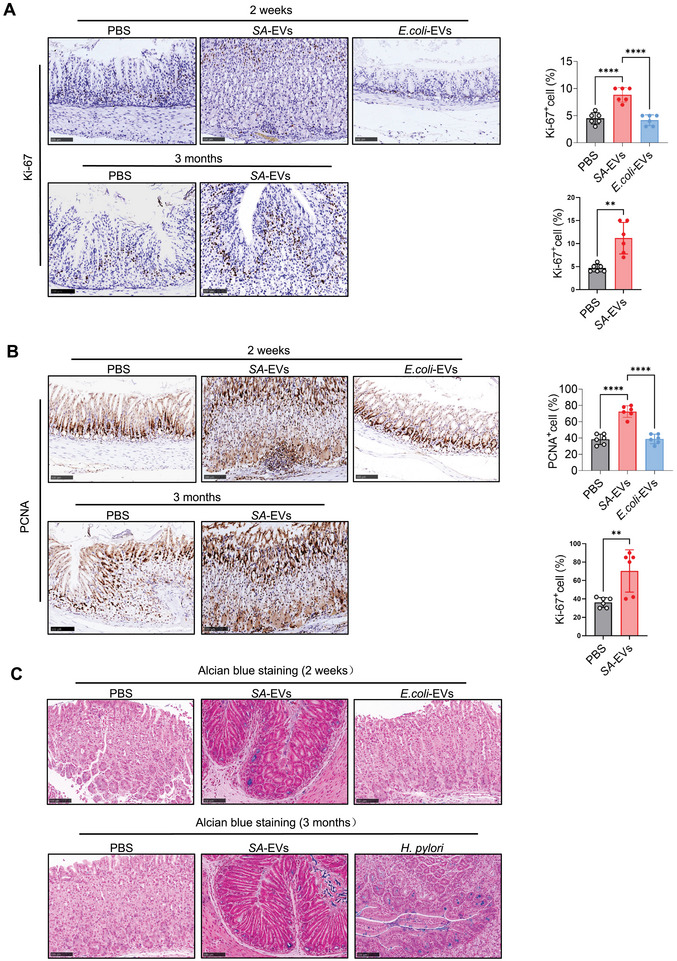
*SA*‐EVs promote gastric epithelial cell proliferation. (A,B) At 2 weeks and 3 months post‐infection, the expression levels of the cell proliferation markers Ki‐67(A) and proliferating cell nuclear antigen (PCNA) (B) as well as gastric tissue proliferation, were elevated in mice treated with *SA*‐EVs. Scale bars represent 100 µm. (C) Alcian blue staining in mice treated with *SA*‐EVs, PBS, E.coli‐EVs, *H. pylori*. Scale bars represent 100 µm. Data is shown as mean ± SD, with dots representing individual donors (average of technical duplicates, *n* = 6). Statistical differences between groups were determined using one‐way ANOVA with Tukey post‐tests. *p* < 0.05 (*); *p* < 0.01 (**); *p* < 0.001 (***); *p* < 0.0001 (****); ns: not significant.

**FIGURE 5:**
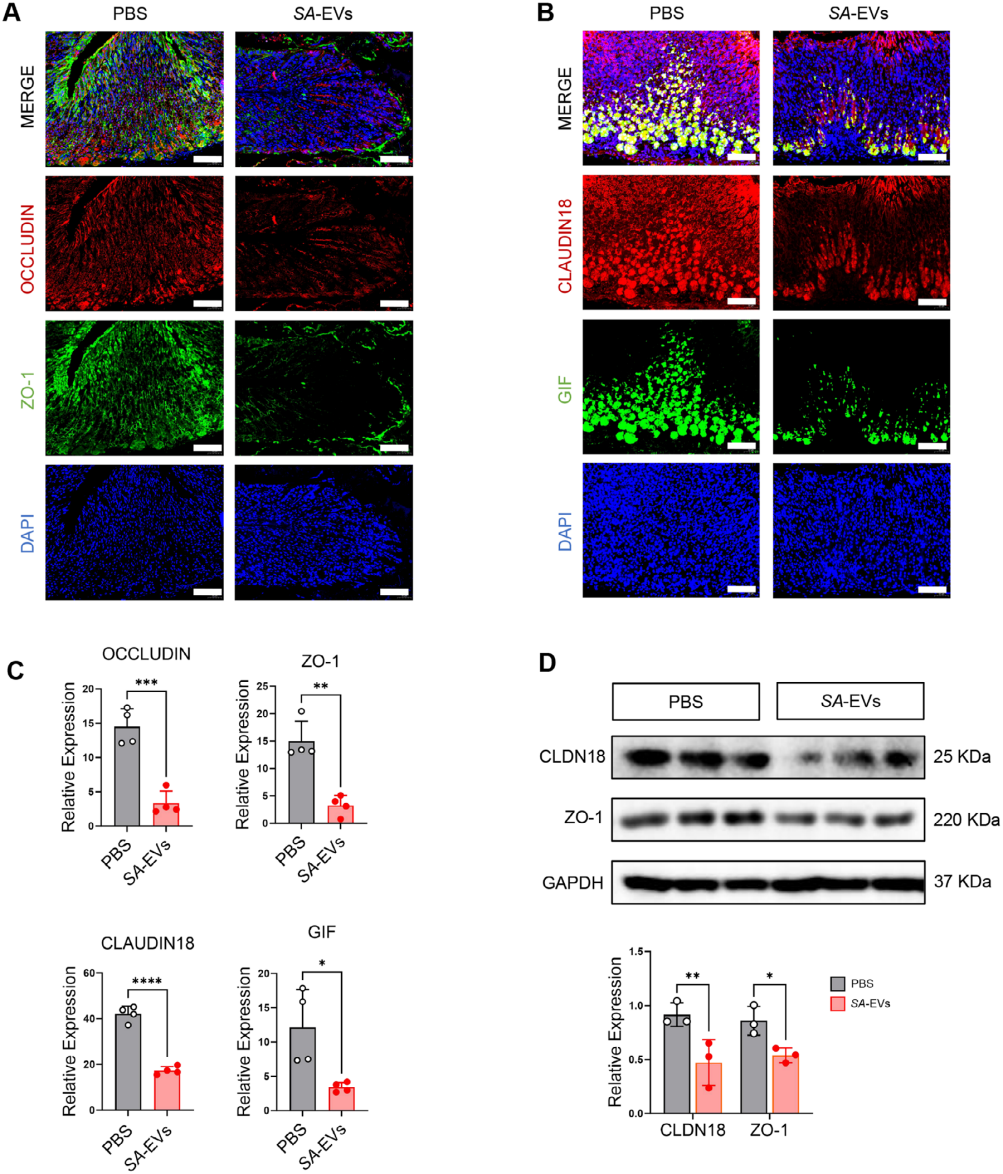
*SA*‐EVs disrupt gastric barrier integrity. (A)The expression of Zonula occludens(ZO‐1) and gastric tight junction markers (OCCLUDIN) in *SA*‐EVs‐treated mice compared with PBS broth control at 3 months post‐infection. Scale bars=50 µm. (B) The expression of gastric intrinsic factor (GIF) and gastric tight junction markers Claudin18 (CLDN18) in *SA*‐EVs‐treated mice compared with PBS broth control at 3 months post‐infection. Scale bars=50 µm. (C) Quantitative analysis of OCCLUDIN, ZO‐1, CLAUDIN18, and GIF expression is presented as mean±SD, with individual donor data represented by dots (averaged across technical duplicates). (D) Gastric tight junction markers Claudin18 (CLDN18) and Zonula occludens (ZO‐1) were downregulated was verified by Western Blot in *SA*‐EVs‐treated mice compared with PBS control at 3 months post‐infection. Data is shown as mean ± SD, with dots representing individual donors (average of technical duplicates, *n* = 6). Statistical differences between groups were determined using one‐way ANOVA with Tukey post‐tests. *p* < 0.05 (*); *p* < 0.01 (**); *p* < 0.001 (***); *p* < 0.0001 (****); ns: not significant.

The mucosal barrier encompasses the structural and functional components that prevent toxins and pathogenic bacteria from penetrating mucous membranes and invading tissues, organs, and the circulatory system [32]. Gastritis is typically associated with the disruption of the gastric mucosal barrier [33], and restoring barrier integrity while suppressing inflammation is considered a key therapeutic strategy [34]. We further investigated the effects of *SA*‐EVs on gastric barrier function and microbiota. *SA*‐EVs promoted epithelial cell proliferation, as evidenced by increased Ki‐67 (Figure 4A) and PCNA expression (Figure 4B), but had no significant effect on inhibiting apoptosis (Figure S2A). Tight junction proteins, including Claudin‐18 (CLDN18), Occludin, and ZO‐1, which are specifically expressed in the stomach, were significantly disrupted in the neck region of the stomach body and the base of the antrum three months post‐infection with *SA*‐EVs, as shown by immunofluorescence staining (Figure 5A–C). Western blot analysis of the aforementioned tight‐junction proteins further confirmed that *SA*‐EVs infection reduced their expression levels (Figure 5D). Histological analysis further confirmed these effects, revealing an increase in Alcian blue‐positive cells (Figure 4C) and a decrease in gastric intrinsic factor (GIF)‐positive parietal cells (Figure 5B). Together, these findings indicate that *SA‐*EVs impair epithelial integrity and disrupt mucosal barrier function, potentially exacerbating the progression of gastritis.

### 2.5 SA‐EVs Proteome Contains Virulence Factors TMPC and FBP62 and Activates MAPK Signaling

**FIGURE 6:**
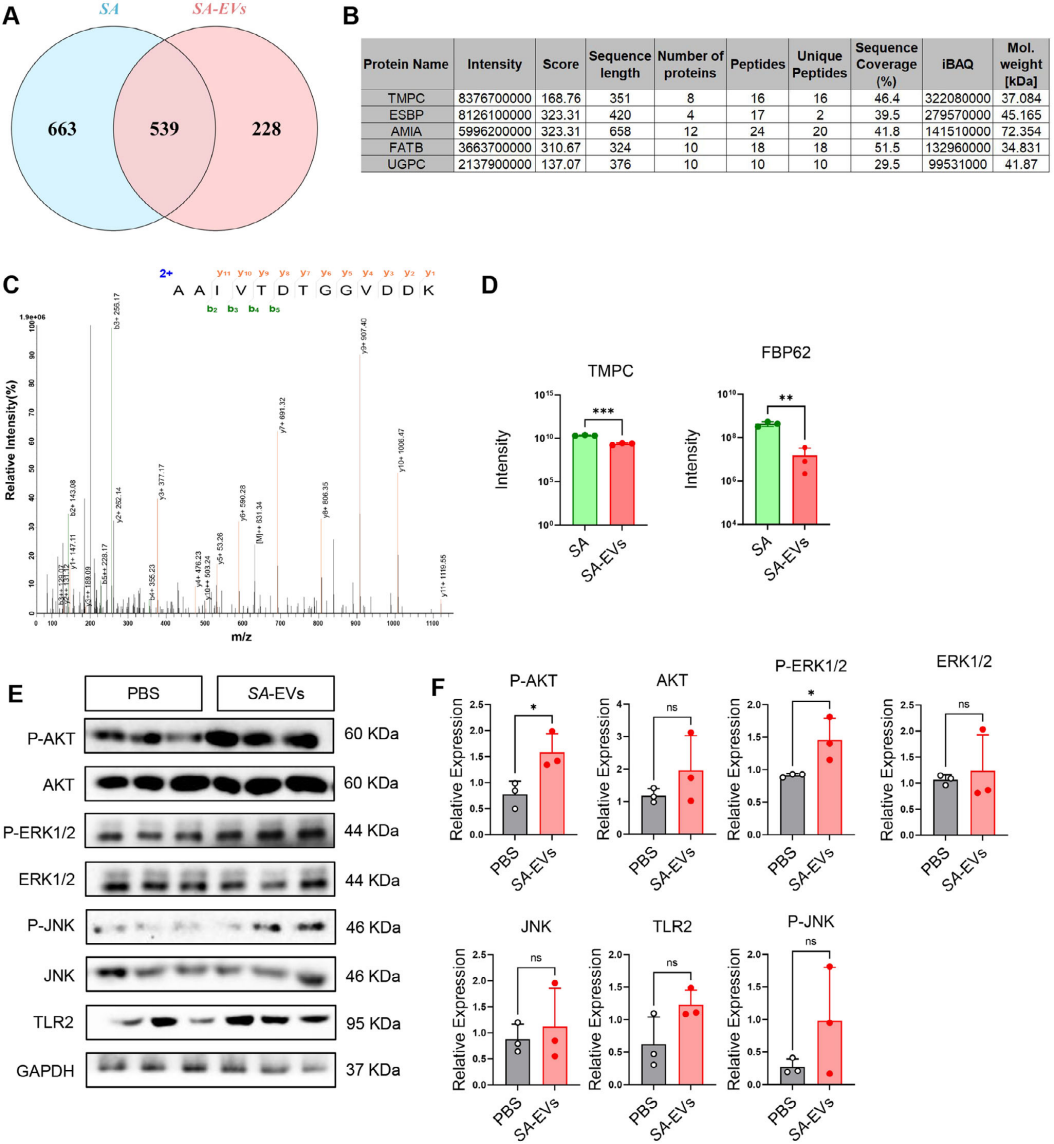
*SA*‐EVs contain TMPC and FBP62 and activate MAPK signaling. (A) Venn diagram illustrating the comparison between *SA* and *SA*‐EVs. (B) List of candidates with top 5 scores in LC‐MS/MS analysis of *SA*‐EVs. (C) Secondary map of TMPC in *SA*‐EVs. (D) The relative levels of TMPC and FBP62 proteins in *SA* and *SA*‐EVs. (E‐F) Western blot analysis(E) and quantitative analysis(F) of the activation of the MAPK pathway in *SA*‐EVs‐treated mice compared with the PBS control group of BALB/c mice at 3 months post‐infection, with increased p‐ERK, p‐JNK, and p‐AKT, and TLR2 (*n* = 6 per group). Data is shown as mean ± SD, with dots representing individual donors (average of technical duplicates, *n* = 6). Statistical differences between groups were determined using one‐way ANOVA with Tukey post‐tests. *p* < 0.05 (*); *p* < 0.01 (**); *p* < 0.001 (***); *p* < 0.0001 (****); ns: not significant.

Previous studies have shown that *SA* interacts with gastric mucosal epithelial cells through TMPC, activating MAPK signaling and promoting the development of gastritis and, in some cases, gastric cancer [5]. Liquid chromatography‐tandem mass spectrometry (LC‐MS/MS) analysis identified 539 common proteins in both *SA* and *SA*‐EVs (Figure 6A), with TMPC being the most abundant among the top five proteins detected (Figure 6B–D and Table S1, Data are shown in Supporting datasheet 1). Moreover, Western blot experiments confirmed a significant increase in the protein expression of p‐ERK1/2, p‐JNK, and p‐AKT in *SA*‐EVs‐treated mice (Figure 6E,F), suggesting that *SA*‐EVs may serve as vectors for delivering TMPC to target cells. Additionally, studies have identified FBP62 as a virulence factor of *SA*. We also detected this protein in *SA*‐EVs (Figure 6D); however, due to the lack of specific antibodies, we were unable to perform a detailed analysis. Furthermore, previous research has shown that *SA* exerts its pathogenic effects through TLR2 [35]. We also performed Western blot analysis to validate the *SA*‐EVs‐treated group (Figure 6E,F).

By transferring TMPC, FBP62, and potentially other virulence factors to gastric epithelial cells, *SA*‐EVs may mimic or enhance the bacterium's ability to disrupt the gastric mucosal barrier, induce inflammation, and accelerate the progression of gastritis. These findings underscore the critical role of *SA*‐EVs in the pathogenesis of gastritis and highlight their importance as a key focus for further investigation.

### 2.6 SA‐EVs Trigger Macrophage‐Centered Cytokine Signaling in the Stomach

**FIGURE 7:**
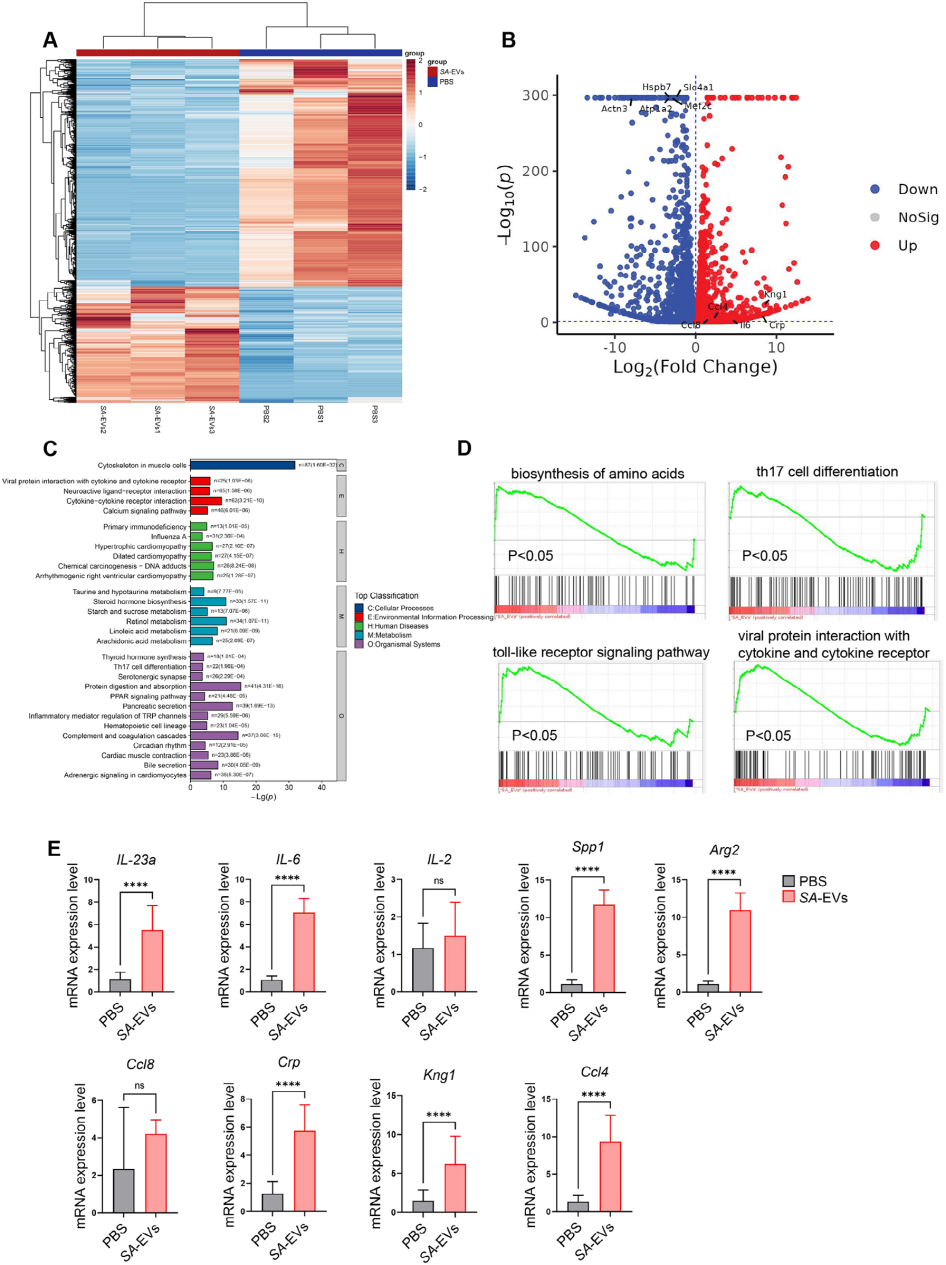
Transcriptomic analysis reveals inflammatory signatures in *SA*‐EVs‐treated gastric tissues. (A) After intragastric administration of *SA*‐EVs to mice for 3 months, the gastric tissues of the mice were taken for transcriptomic analysis. The differential gene volcano map indicates the upregulation of inflammation‐related gene expression (*n* = 3 replicates). (B) Cluster analysis was conducted on the expression of differential genes in the gastric tissues of mice intragastric with *SA*‐EVs or PBS (*n* = 3 replicates). (C) In the gastric tissues of mice with intragastric administration of *SA*‐EVs, the cytokine‐cytokine receptor interaction pathway was enriched. (D) The GSEA map of the related inflammatory pathways. (E) The gastric tissues of mice intragastric with *SA*‐EVs or PBS were verified by q‐pcr. Data is shown as mean ± SD, with dots representing individual donors (average of technical duplicates, *n* = 6). Statistical differences between groups were determined using one‐way ANOVA with Tukey post‐tests. *p* < 0.05 (*); *p* < 0.01 (**); *p* < 0.001 (***); *p* < 0.0001 (****); ns: not significant.

To further investigate the molecular mechanisms underlying the gastric inflammation induced by *SA*‐EVs, we conducted transcriptomic analysis of mouse stomach tissues after 3 months of repeated intragastric administration. The transcriptomic analysis showed that there were significant differences in gene expression between the *SA*‐EVs group and the PBS group (Figure 7A). It is notable that some genes related to inflammation and regulation of macrophage polarization showed significant upregulation, including *IL‐23a, IL‐6, IL‐2, Ccl8, Ccl4, Crp, Kng1, Spp1*, and *Arg2* (Figure 7B; Table S2, Data are shown in Supporting datasheet 2). These genes are known to be critically involved in promoting pro‐inflammatory signaling and modulating macrophage phenotypes. Specifically, *Spp1* and *Arg2* are closely linked to macrophage activation and polarization [36, 37], suggesting a dynamic macrophage response contributing to the observed gastritis phenotype. These transcriptomic findings were further validated by quantitative PCR (qPCR), which confirmed the significant upregulation of these key inflammatory and macrophage‐related genes in the gastric tissue of *SA*‐EVs‐treated mice (Figure 7E; Table S7, Data are shown in Supporting datasheet 7). Moreover, flow cytometry analysis of gastric tissues further revealed a significant increase in macrophages in the *SA*‐EVs group, accompanied by elevated expression of CD206 and F4/80 (Figure S7C–E). Further pathway enrichment analysis based on the differentially expressed genes indicated an activation of cytokine‐related signaling pathways, particularly Cytokine‐ cytokine receptor interaction and Th17 cell differentiation (Figure 7C,D; Table S4A,B, Table S3, Data are shown in Supporting datasheet 3). This transcriptomic evidence is consistent with the histopathological observations of increased immune cell infiltration and disruption of gastric epithelial integrity.

### 2.7 SA‐EVs Reshape the Fecal Metabolome and Gut Microbial Communities

**FIGURE 8:**
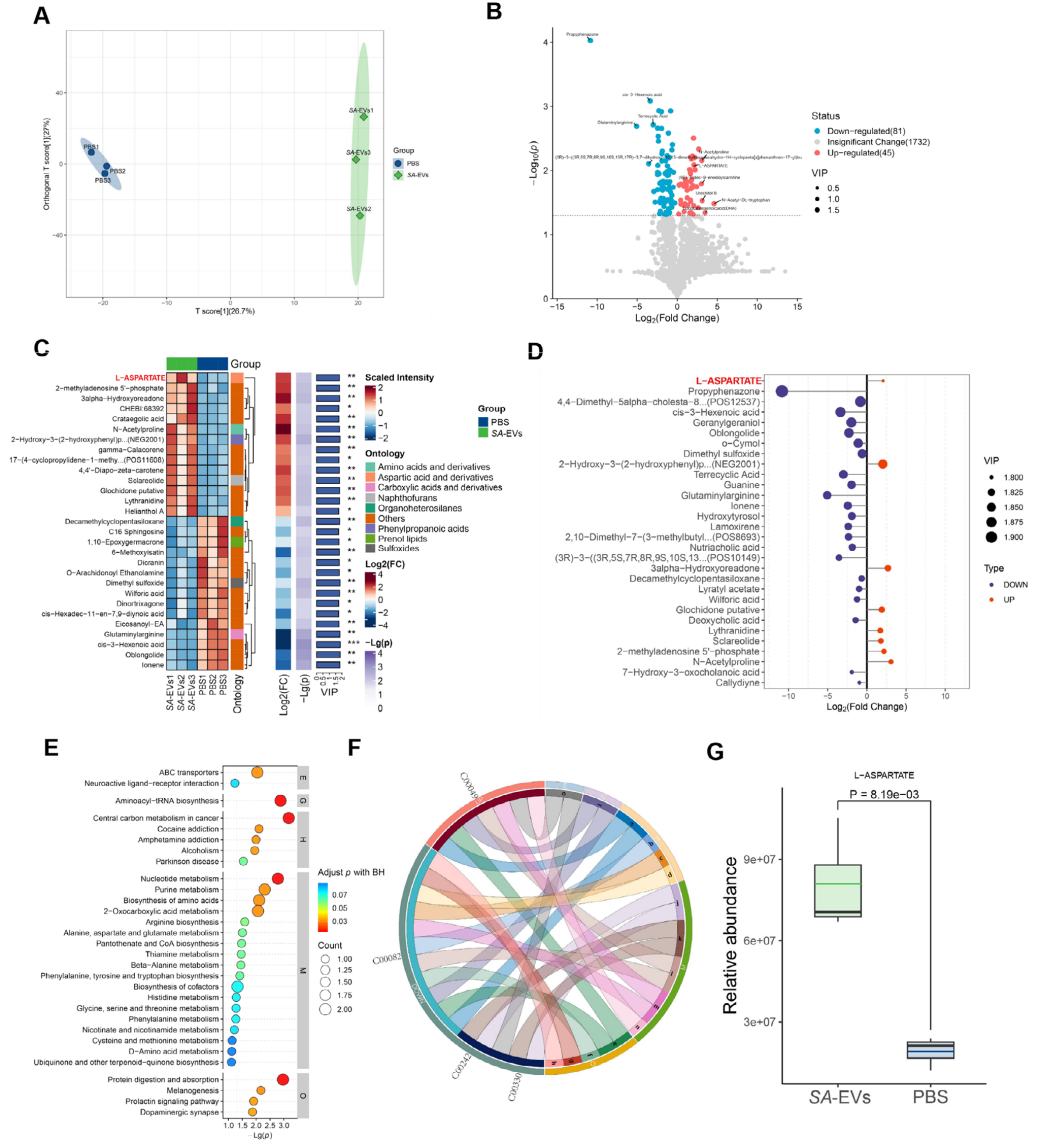
*SA*‐EVs alter host fecal metabolites, including aspartate enrichment. (A) OPLS‐DA indicated the differences in fecal metabolites between mice gavage *SA*‐EVs and PBS. (B) The volcano plot shows the differences in enriched metabolites in the feces of gavage *SA*‐EVs and PBS mice. (C,D) Heat maps (C) and bubble maps (D) suggest the differences in metabolite enrichment. (E,F) Bubble chart(E) and Chord Diagram (F) of metabolic pathways. (G) Relative levels of aspartate in the feces of mice treated with *SA*‐EVs and PBS. Data is shown as mean ± SD, with dots representing individual donors (average of technical duplicates, *n* = 6). Statistical differences between groups were determined using one‐way ANOVA with Tukey post‐tests. *p* < 0.05 (*); *p* < 0.01 (**); *p* < 0.001 (***); *p* < 0.0001 (****); ns: not significant.

Fecal metabolome analysis revealed significant differences in the fecal metabolites of mice treated with *SA*‐EVs compared to the PBS group (Figure 8A). A volcano plot identified 45 upregulated and 81 downregulated metabolites, many of which are closely associated with inflammation, oxidative stress, and gut microbiota imbalances—key characteristics of gastritis (Figure 8B). Notably, aspartate levels were significantly elevated (Figure 8C,D; Table S4, Data are shown in Supporting datasheet 4), aligning with recent findings that *Salmonella* Typhimurium can proliferate in an aspartate‐dependent manner, thereby playing a crucial role in inflammation [38]. In addition, other inflammation‐related metabolites, such as 2‐Hydroxy‐3‐(2‐hydroxyphenyl) propanoic acid and Glochidone putative, exhibited significant alterations. Metabolic pathway enrichment analysis further identified disruptions in pathways linked to gastritis, including inflammatory factor dysregulation, nucleic acid metabolism imbalance, oxidative stress, and neurotransmitter regulation. Specifically, key pathways such as alanine, aspartate, and glutamate metabolism, arginine biosynthesis, and dopaminergic synapse function were notably affected (Figure 8E,F; Figure S5A–C and Table S5, Data are shown in Supporting datasheet 5). Importantly, fecal samples from *SA*‐EVs‐treated mice showed a significant increase in aspartate levels (Figure 8G), with enrichment in alanine, aspartate, and glutamate metabolism (Figure 8E). These findings suggest that these metabolic changes may serve as potential biomarkers for the onset and progression of gastritis.

To better understand the significance of metabolic changes in mouse feces, we performed an integrative analysis combining gastric transcriptomics with non‐targeted fecal metabolomics. Among the commonly enriched pathways, protein digestion and absorption and metabolic pathways were significantly upregulated (Figure S6A,B), further suggesting that alterations in aspartate metabolism may play a critical role in the inflammatory response. Moreover, aspartate serves as a key metabolic node in the tricarboxylic acid (TCA) cycle and plays a vital role in energy metabolism, amino acid biosynthesis, and nitrogen balance through its cross‐regulation with other metabolic pathways [39]. Elevated levels of aspartate are associated with significant changes in gene expression in the mouse stomach (Figure S6C). Within its complex metabolic network, the expression levels of *Agxt*, *Gls2*, and *Cps1* are notably upregulated (Figure S6D), with statistical analyses confirming their differential expression (Figure S6E–G). Our data implicates aspartate metabolism in the progression of gastritis. Although the number of enriched genes is modest, the pronounced changes observed suggest that this pathway may serve as a critical regulatory point with broad influence on gastric inflammation.

**FIGURE 9:**
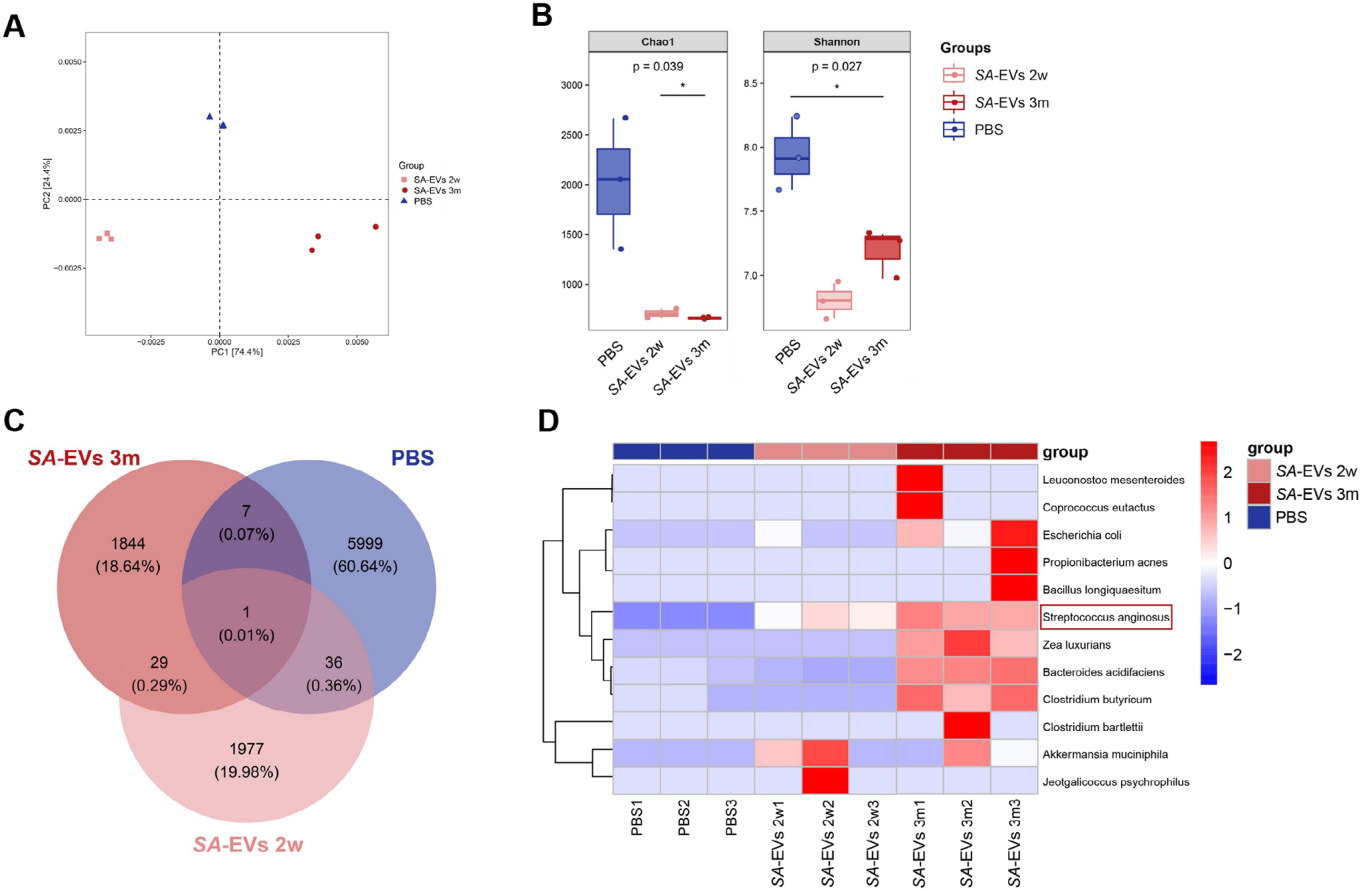
*SA* colonization and gut microbiota alterations induced by *SA*‐EVs. (A) After gavage of *SA*‐EVs for two weeks and three months, the PCA of fecal microbiota differences compared with the PBS group. (B) Chao1 and Shannon demonstrated the corresponding alpha diversity indices. (C) The Venn diagram shows the number of overlapping microbiota among the three groups. (D) The heat map shows the relevant differential flora. Data is shown as mean ± SD, with dots representing individual donors (average of technical duplicates, *n* = 6). Statistical differences between groups were determined using one‐way ANOVA with Tukey post‐tests. *p* < 0.05 (*); *p* < 0.01 (**); *p* < 0.001 (***); *p* < 0.0001 (****); ns: not significant.

What's more, to evaluate whether the administered vesicles influenced gut microbial composition or supported microbial persistence, we conducted *16S rRNA* sequencing on fecal samples collected from mice after prolonged EV administration. In addition to showing the differences in the composition of the intestinal microbiota (as shown in the isolated graph in the principal coordinate analysis (PCoA), (Figure 9A) and richness (such as lower Chao1 and Shannon indices, Figure 9B) between in gastric *SA*‐EVs and PBS mice, we also observed significant alterations in the relative abundances of specific microbial taxa in the *SA*‐EVs group compared to the PBS group (Figure 9C,D). Notably, the abundance of *SA* was higher in the *SA*‐EVs 3months group than in the *SA*‐EVs 2weeks group (Table S6, Data are shown in Supporting datasheet 6). Fecal samples from mice administered PBS or *SA*‐EVs for 2 weeks or 3 months were analyzed by qPCR, further confirming this finding (Figure S7B). Taken together, these findings, the shift in overall microbial structure, the reduction in community richness, and the specific alterations in key taxa, suggest that *SA*‐EVs do not merely alter but systemically reprogram the gut microbiota. This microbiota dysbiosis, in concert with the direct local effects of *SA*‐EVs on the gastric mucosa, likely creates a pro‐inflammatory environment that fuels the progression of gastritis.

### 2.8 Knockout of the Tmpc and Fbp62 Genes in SA Significantly Reduced its Pathogenicity

**FIGURE 10:**
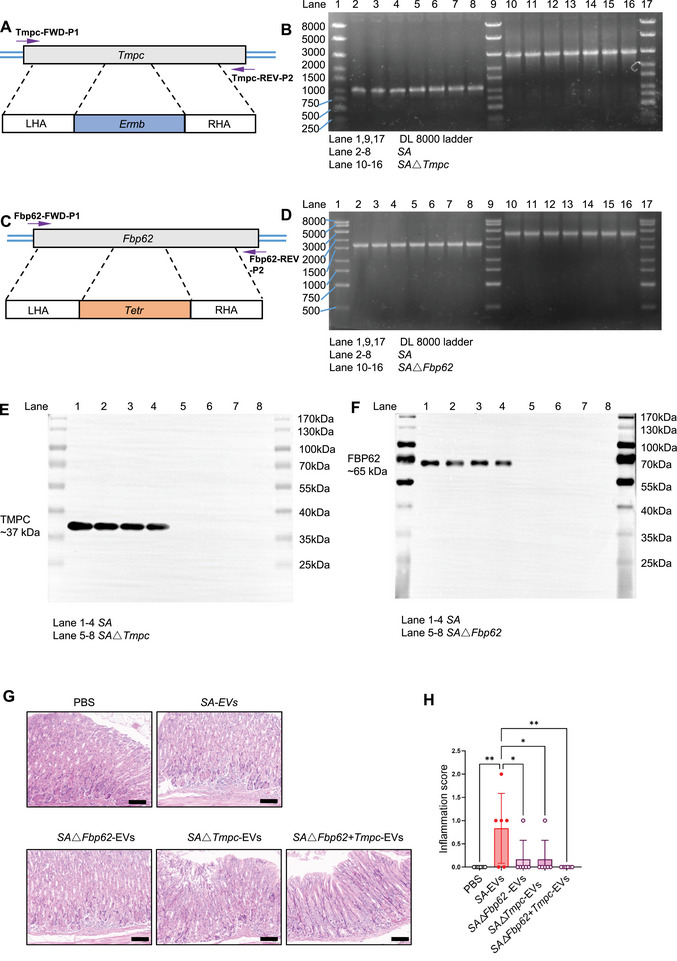
TMPC and FBP62 are critical mediators of the pathogenic activity of *SA*‐EVs. (A) The schematic illustrates the insertional inactivation of Tmpc in *SA*. The plasmid *Ermb* was constructed and introduced into S*A* to mediate homologous recombination. (B) PCR detection of the Tmpc gene in *SA* and *SA*△*Tmpc* strains. *n* = 6 for each group. (C) The schematic illustrates the insertional inactivation of Fbp62 in S*A*. The plasmid *Tetr* was constructed and introduced into *SA* to mediate homologous recombination. (D) PCR detection of the *Fbp62* gene in *SA* and *SA*△*Fbp62* strains. *n* = 6 for each group. (E) Analysis of TMPC protein levels in SA and SA△*Tmpc*. *n* = 4 for each group. (F) Analysis of *Fbp62* protein levels in SA and SA△*Fbp62*. *n* = 4 for each group. (G) Representative hematoxylin and eosin (H&E) staining images of stomach tissue from mice treated with *SA*‐EVs, PBS, *SA*△*Fbp62*‐EVs, *SA*△*Tmpc*‐EVs, and *SA*△*Fbp62*+*Tmpc*‐EVs at two weeks post‐infection. Scale bars = 50 µm. (H) Gastric inflammation scores for the different groups: score 0, no inflammation; score 1, mild inflammation; score 2, moderate inflammation; score 3, severe inflammation; score 4, critical inflammation. Data is shown as mean ± SD, with dots representing individual donors (average of technical duplicates, *n* = 6). Statistical differences between groups were determined using one‐way ANOVA with Tukey post‐tests. *p* < 0.05 (*); *p* < 0.01 (**); *p* < 0.001 (***); *p* < 0.0001 (****); ns: not significant.

In our earlier experiments, proteomic analysis revealed that *SA*‐EVs are enriched in two prominent virulence‐associated proteins, TMPC and FBP62. To clarify their functional contributions to EV‐mediated gastric pathology, we generated *SA* mutant strains lacking either Tmpc or Fbp62, as well as a double‐knockout strain lacking both genes (Figure 10A,C). The wild‐type Tmpc and Fbp62 fragments measured 1.02 and 1.62 kb (Figure S8A,C), respectively. Electrophoresis of the engineered strains confirmed successful gene disruption, yielding fragments of approximately 2.8 kb for △Tmpc and 2.5 kb for △*Fbp62* (Figure 10C,D, and Figure S8B,D). Western blot analysis further validated the loss of TMPC and FBP62 proteins in the corresponding mutants (Figure 10E,F). The extracellular vesicles from the △*Tmpc* and △*Fbp62 SA* were isolated and purified as the same wit WT *SA*‐EVs. The TEM image is shown in Figure S9.

**FIGURE 11:**
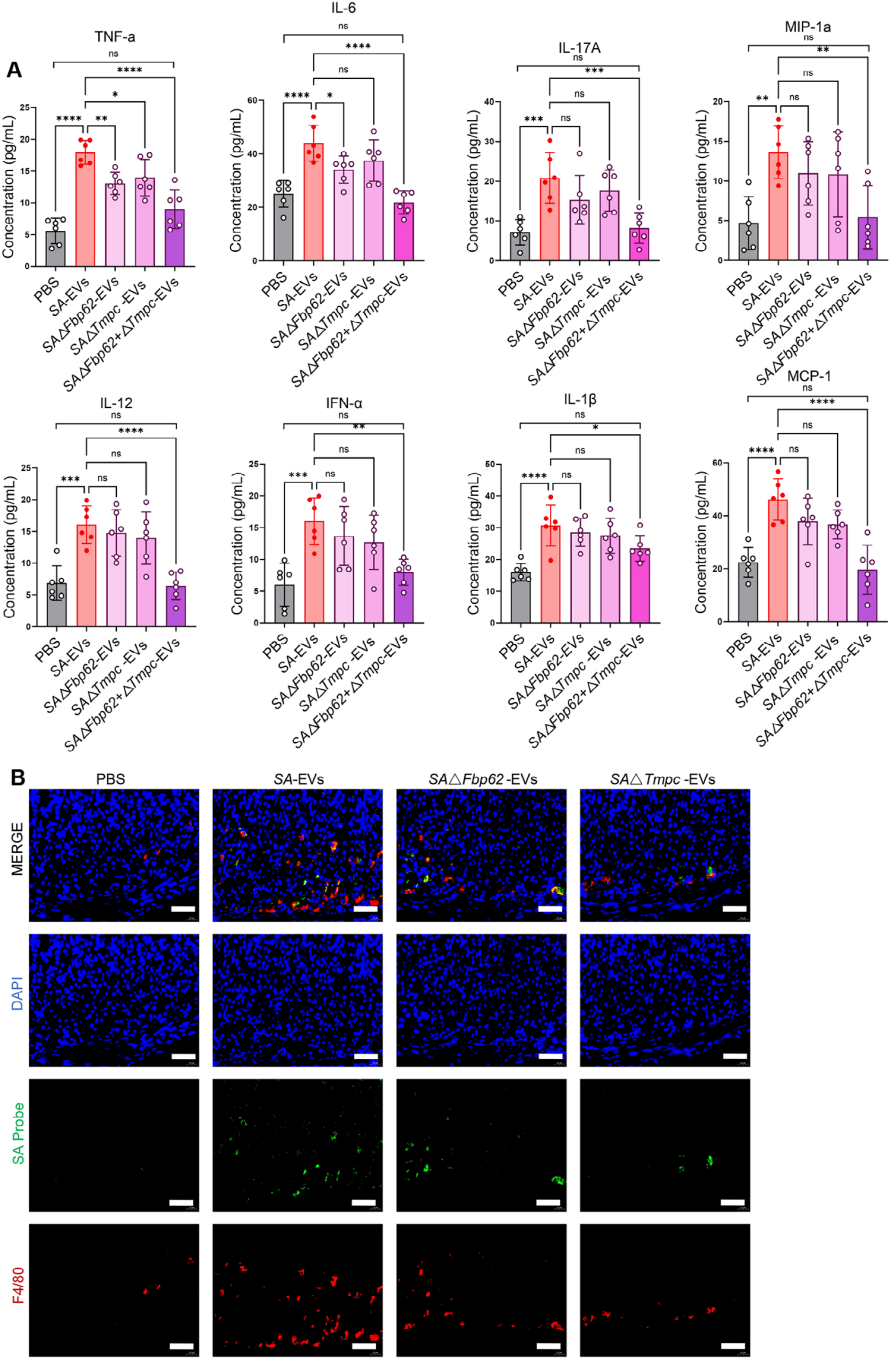
The pathogenicity of *SA*‐EVs lacking TMPC and FBP62 is significantly reduced. (A) Serum levels of TNF‐α, IL‐6, IL‐17, MIP‐a, IL‐12, IFN‐a, IL‐1β, and MCP‐1 were upregulated following infection at two weeks post‐infection. (B) Two weeks after intragastric administration, the expression levels of *SA* and F4/80 in gastric tissues were assessed across the different treatment groups. Scale bars = 50 µm. Data is shown as mean ± SD, with dots representing individual donors (average of technical duplicates, *n* = 6). Statistical differences between groups were determined using one‐way ANOVA with Tukey post‐tests. *p* < 0.05 (*); *p* < 0.01 (**); *p* < 0.001 (***); *p* < 0.0001 (****); ns: not significant.

To assess the pathogenic relevance of these virulence factors, mice were intragastrically administered EVs derived from the mutant strains. After two weeks, H&E staining revealed that EVs lacking TMPC or FBP62, individually or in combination, induced markedly attenuated gastric pathology compared with wild‐type *SA*‐EVs (Figure 10G,H). Consistent with these histological improvements, serum concentrations of pro‐inflammatory cytokines, including TNF‐α, IL‐6, IL‐17A, MIP‐1a, IL‐12, IFN‐a, IL‐1β, and MCP‐1 were substantially reduced in mice treated with knockout‐derived EVs (Figure 11A). Immunofluorescence staining further demonstrated diminished gastric enrichment of SA in knockout groups and a significant reduction in F4/80^+^ macrophage infiltration, which was otherwise pronounced in the *SA*‐EVs group (Figure 11B).

Collectively, these findings demonstrate that TMPC and FBP62 are key contributors to the pathogenicity of *SA*‐EVs. Loss of either protein, particularly in the double‐knockout strain, substantially diminishes the ability of *SA*‐EVs to trigger macrophage activation, cytokine production, and gastric tissue injury. This highlights the essential role of these EV‐carried virulence factors in driving *SA*‐mediated gastritis.

## 3 Discussion

Gastric inflammation has classically been attributed to *H. pylori*, which infects over half the world's population and orchestrates chronic gastritis, mucosal atrophy, and even cancer through a suite of virulence factors [33]. However, growing evidence suggests that other members of the gastric microbiota can also drive disease. Microbiome surveys of gastritis and gastric cancer patients have repeatedly found enrichment of oral‐type organisms, including *Prevotella*, *Rothia*, *Peptostreptococcus*, *Parvimonas, and Streptococcus* (notably *S. anginosus*) in diseased stomachs [33, 40]. For example, Coker et al. identified *S. anginosus* among the top species associated with gastric tumors [7] and Fu et al. recently showed that *S. anginosus* colonizes the mouse stomach and induces acute and chronic gastritis, atrophy, metaplasia, and dysplasia via its surface adhesin (TMPC) engaging the ANXA2–MAPK pathway [5]. These findings redefine our view of gastric pathogenesis, indicating that non‐*H. pylori* pathogens can initiate and sustain inflammation. In this context, our work expands on this understanding by demonstrating that *SA*‐EVs are not passive byproducts of bacterial activity, but rather active mediators capable of disrupting gastric epithelial integrity, promoting inflammatory responses, and remodeling the host immune and metabolic landscape.

Through a combination of in vitro and in vivo experiments, we show that *SA*‐EVs are internalized by gastric epithelial cells and accumulate within the stomach after oral administration. Notably, both acute (2‐week) and chronic (3‐month) exposure to *SA*‐EVs in mice led to the development of gastritis, as evidenced by histological analysis, inflammatory cell infiltration, and upregulation of key cytokines such as IL‐6, IL‐17A, TNF‐α, and CCL8. Importantly, chronic administration resulted in sustained immune activation and structural deterioration of the gastric barrier, as indicated by the disruption of tight junction proteins including Claudin‐18, Occludin, and ZO‐1. Disruption of tight junctions is known to increase mucosal permeability and allow microbial translocation, indeed, similar barrier loss is observed in *H. pylori* gastritis [33]. For example, Wei et al. found that macrophage‐derived CCL3 (from *H. pylori* infection) can directly abrogate gastric epithelial tight junctions. Our observation that *SA*‐EVs alone cause junctional disruption suggests that vesicle‐carried factors trigger a cascade of immune signaling and tissue damage [33]. In sum, *SA*‐EVs by themselves suffice to initiate both acute and chronic gastritis in vivo, establishing these vesicles as active pathogenic agents. These proteins are essential for maintaining epithelial integrity, and their loss is associated with increased permeability and enhanced susceptibility to bacterial invasion [41, 42, 43]. Such structural disruption likely contributes to the persistence of inflammation and may create a favorable niche for microbial persistence or secondary infections.

One of the key findings of this study is the identification of TMPC and FBP62—two virulence‐associated proteins previously implicated in *SA* pathogenicity—within the protein cargo of *SA*‐EVs [5, 13]. Proteomic analysis confirmed their abundance in purified vesicle preparations. TMPC, in particular, has been shown to bind annexin A2 and activate downstream MAPK signaling, leading to epithelial proliferation, inflammation, and even oncogenic transformation in gastric tissues [5]. Our results demonstrate that *SA*‐EVs deliver TMPC to gastric cells, as evidenced by increased phosphorylation of ERK1/2, JNK, and AKT, supporting the notion that vesicle‐mediated delivery of virulence factors contributes directly to host cell signaling alterations. While FBP62 could not be further characterized due to antibody limitations, its presence in the EVs cargo suggests a potential role in enhancing bacterial adhesion or immune evasion. These findings underscore the similarity between *SA*‐EVs and EVs from other gastric pathogens, such as *H. pylori*, whose EVs carry pathogenic proteins like CagA and VacA [26, 44, 45].

Importantly, our gene‐knockout experiments provide functional validation that TMPC and FBP62 are not only present in *SA*‐EVs but are essential mediators of *SA*‐EVs pathogenicity. We constructed *SA* strains lacking *Tmpc*, *Fbp62*, or both genes, confirmed successful deletions through PCR and Western blot, and isolated their corresponding EVs. Remarkably, EVs derived from knockout strains exhibited significantly diminished pathogenicity in vivo. Mice receiving *SA*Δ*Tmpc*‐EVs, *SA*Δ*Fbp62*‐EVs, or *SA*Δ*Tmpc+Fbp62*‐EVs displayed markedly reduced gastric pathology, drastically lower cytokine levels (including TNF‐α, IL‐6, and IL‐17A), and decreased *SA* enrichment in gastric tissues. In parallel, the elevated F4/80^+^ macrophage infiltration observed in wild‐type *SA*‐EVs‐treated mice was significantly attenuated following gene deletion. These findings provide direct causal evidence that TMPC and FBP62 are key EV‐associated virulence factors driving epithelial injury, inflammatory activation, and macrophage polarization. The knockout data, therefore, functionally anchor the mechanistic model proposed in this study and confirm that *SA*‐EVs–mediated gastritis depends on discrete and identifiable molecular effectors rather than non‐specific microbial components.

Beyond structural and proteomic insights, transcriptomic analysis of mouse gastric tissue revealed profound alterations in gene expression following chronic *SA*‐EVs exposure. Specifically, we observed upregulation of multiple genes associated with inflammation and macrophage polarization, including *IL‐6*, *Ccl8*, *Ccl4*, *Spp1*, and *Arg2*. These genes are not only indicative of an activated immune state but also suggest dynamic involvement of macrophage subtypes in disease progression [46, 47]. *Spp1* is known to function as a chemotactic factor and immune modulator, particularly in chronic inflammatory settings, while *Arg2* plays a central role in regulating nitric oxide metabolism and is often upregulated in alternatively activated (M2‐like) macrophages [36, 37, 48]. The concurrent increase in both genes points to a complex interplay between pro‐inflammatory and tissue‐repairing macrophage phenotypes, which may underlie the chronicity of the observed inflammation. Furthermore, cytokine‐cytokine receptor interaction and Th17 cell differentiation pathways were significantly enriched in the *SA*‐EVs group. Previous studies have shown glutamine metabolism can promote the differentiation of Th17 cells [49]. There are also studies reporting that downstream metabolites of glutamic acid can promote Th17 cell differentiation, and that targeting glutamate‐dependent metabolic pathways may suppress Th17 differentiation, thereby modulating the Th17/iTreg balance to alleviate autoimmune diseases [50]. In our model, *SA*‐EVs significantly altered amino acid and energy metabolism (as discussed below), which may in turn facilitate Th17‐driven responses. In sum, *SA*‐EVs exposure appears to trigger a sustained inflammatory program involving macrophage recruitment and polarization, chemokine cascades, and T cell subsets characteristic of chronic gastritis.

We observed that *SA*‐EVs exert effects beyond the gastric mucosa, influencing gut microbial communities and metabolism. *16S rRNA* sequencing of feces revealed detectable *SA* DNA only in *SA*‐EVs–treated mice, suggesting that EVs might promote survival or colonization of the parent bacterium in the gastrointestinal tract. This observation raises the possibility that *SA*‐EVs not only affect host immunity but may also facilitate the colonization or persistence of their parent bacterium in the gastrointestinal tract. Whether this reflects direct vesicle‐mediated protection of bacterial DNA, modulation of mucosal immunity to favor *SA*, or promotion of a microbial niche conducive to its growth remains to be further investigated. This raises intriguing possibilities: *SA*‐EVs could protect bacterial DNA in the gut, or they might modulate the mucosal immune environment (e.g. via barrier disruption or cytokine secretion) to favor *S. anginosus* persistence. In any case, the data indicate that *SA*‐EVs contribute to a gut niche that supports their producer, a hallmark of chronic infection.

The gut microbiota contributes to the regulation of various host metabolic pathways [51]. These metabolic processes—including glycolysis, oxidative phosphorylation, and fatty acid oxidation—can influence the phenotype and function of immune cells, thereby playing a crucial role in modulating inflammatory responses [52, 53, 54, 55]. Studies have demonstrated that bifidobacteria can effectively prevent hepatic steatosis and inflammation through the production of indole‐3‐acetic acid [56]. Similarly, Lu et al. found that agmatine, a metabolite derived from the intestinal microbiota, can induce the upregulation of pro‐inflammatory cytokines, thereby promoting intestinal epithelial dysplasia and lymphocytic inflammatory infiltration [57]. Similarly, *Akkermansia muciniphila* has been shown to promote retinoic acid synthesis by dendritic cells, thereby enhancing IL‐22 activity and alleviating colitis in mice [58]. Metabolomic analysis of fecal samples provided additional insights into the systemic impact of *SA*‐EVs. Notably, we observed a significant elevation in aspartate levels and enrichment of metabolic pathways associated with amino acid biosynthesis, oxidative stress, and neurotransmitter signaling. Aspartate serves as a central hub in cellular metabolism, contributing to the tricarboxylic acid (TCA) cycle, nucleotide biosynthesis, and the maintenance of nitrogen balance [39]. Aspartate has recently been implicated in supporting the growth of enteric pathogens such as *Salmonella* Typhimurium, and its elevation may signal broader metabolic dysregulation conducive to inflammation [38]. Our findings suggest that similar metabolic dependencies may underlie *SA*‐EVs‐mediated gastric inflammation. Notably, elevated aspartate levels were associated with altered expression of *Agxt*, *Gls2*, and *Cps1* in gastric tissues, supporting the hypothesis that metabolic reprogramming contributes to immune modulation and tissue pathology. Other altered metabolites, such as 2‐hydroxy‐3‐(2‐hydroxyphenyl) propanoic acid, are associated with oxidative stress and microbial metabolism, further highlighting the metabolic consequences of *SA*‐EVs exposure. Together, these findings suggest that *SA*‐EVs instigate a feed‐forward loop: they induce local inflammation and barrier breach, which perturbs the microbiome and metabolome, further amplifying immune activation and pathogen persistence.

Despite the strength of these findings, our study has several limitations. First, although murine models provide valuable insights into host–pathogen interactions, they may not fully recapitulate human gastric physiology or immune responses. Future studies utilizing human gastric organoids, ex vivo tissue cultures, or patient‐derived biopsy specimens are needed to validate the clinical relevance of our findings. Second, while we identified key virulence factors in *SA*‐EVs, a comprehensive analysis of vesicle contents is warranted to uncover additional bioactive molecules and signaling mediators. Third, the mechanisms underlying the modulation of host microbiota and metabolite profiles by *SA*‐EVs remain speculative. Elucidating the direct versus indirect effects of vesicles on microbial ecology and host metabolism will be critical to fully understanding their pathogenic potential. Finally, the role of host genetic factors, sex differences, and comorbid conditions in shaping responses to *SA*‐EVs warrants further exploration.

Several important avenues for future research arise from this study. Validation of *SA*‐EVs‐mediated gastritis in human clinical samples will be critical to assess translational potential. Identification of EV‐specific biomarkers, such as TMPC, in gastric lavage, stool, or blood could aid in the early diagnosis of non‐*H. pylori*‐associated gastritis. Furthermore, investigating the potential for therapeutic intervention—such as targeting EV production, inhibiting vesicle uptake, or neutralizing key virulence factors—may offer novel strategies for managing *SA*‐associated gastric disease. It is also essential to explore the broader immunological consequences of *SA*‐EVs exposure, including systemic immune modulation and links to extra‐gastrointestinal conditions. Finally, longitudinal studies evaluating the role of *SA*‐EVs in gastric metaplasia or neoplasia could provide critical insights into their contribution to the full spectrum of gastric pathology.

In conclusion, our findings identify *SA*‐EVs as potent modulators of gastric inflammation, capable of driving both acute and chronic gastritis through the delivery of virulence factors, disruption of epithelial barriers, activation of inflammatory pathways, and modulation of gut microbial and metabolic homeostasis. This work broadens the paradigm of bacterial pathogenesis in gastric disease, underscoring the importance of EVs as dynamic vehicles of host‐pathogen interaction. By illuminating the pathogenic potential of *SA* beyond its cellular form, this study provides a foundation for new diagnostic markers and therapeutic approaches targeting bacterial EVs in gastrointestinal disorders.

## 4 Experimental Section/Methods

### 4.1 Reagents

BD BACTO Brain Heart Infusion (BHI broth) (Cat: 237500) was purchased from BD Bioscience (New Jersey, USA). Gram positive bacteria LTA monoclonal antibody (Cat: MA1‐7402), Invitrogen CellMask Deep Red (Cat: C10046) were purchased from Thermo Fisher Scientific (Waltham, Massachusetts, USA). GES‐1 (CL‐0563) was purchased from Probe Store (Guangzhou, China). Mouse‐IL‐6 (Cat: PI326), mouse‐IL‐17A (Cat: PI545), mouse‐IL‐12 (Cat: PI530), mouse‐IL‐22 (Cat: PI591), mouse‐INF‐γ (Cat: PI530), mouse IL‐1β (Cat: PI301), and mouse‐TNF‐α (Cat: PT512) ELISA kit were bought from Beyotime Biotechnology (Shanghai, China). Mouse‐MIP‐1α(Cat: E‐EL‐M0007), mouse‐MCP‐1 (Cat: E‐EL‐M3001), mouse‐CCL‐20 (Cat: E‐EL‐M0013), mouse‐CCL‐8 (Cat: E‐EL‐M3074) ELISA kit were purchased from Elabscience (Shanghai, China). Anti‐mouse IL‐17A antibody (Cat: 506901), anti‐mouse CCL‐20 antibody (Cat: 741299), anti‐mouse TNF‐a antibody (Cat: 506301), anti‐mouse IL‐1β antibody (Cat: 740865), anti‐mouse CCL‐8 antibody (Cat: 741072), anti‐mouse IL‐6 antibody (Cat: 504509), anti‐mouse IL‐10 antibody (Cat: 505029), anti‐mouse IL‐22 antibody (Cat: 740738) were purchased from Biolegend (Guangzhou, China). Anti‐Ki67 Rabbit pAb (Cat: GB111499‐50), Anti‐PCNA Rabbit pAb (Cat: GB11010‐50), TUNEL Apoptosis Detection Kit (CF488 Green fluorescence) (Cat: G1504‐50T), Anti‐ZO1 tight junction protein Rabbit pAb (Cat: GB111981‐50), Anti‐Claudin18 Rabbit pAb (Cat: GB114352‐50), Anti‐Occludin Rabbit pAb (Cat: GB111401‐50) were purchased from BD Servicebio (Guangzhou, China), Akt antibody (Cat: 9272S), Phospho (Ser/Thr) Akt Substrate antibody (Cat: 8611L), p44/42 MAPK (Erk1/2) antibody (Cat: 4668), Phospho‐p44/42 MAPK (Erk1/2) (Thr202/Tyr204) antibody (Cat: 4671), SAPK/JNK antibody (Cat: 9252), phospho‐SAPK/JNK (Thr183/Tyr185) antibody (Cat: 9251), Toll‐like Receptor 2 antibody (Cat: 8206), GAPDH (D4C6R) mouse mAb (Cat: 9251) were purchased from Cell Signaling Technology (Shanghai, China). Invitrogen

### 4.2 Bacteria

S. *anginosus* (BNCC 335670) was obtained from BeNa Culture Collection. *S.anginosus* was cultured on Columbia blood agar and grown in BHI broth (237500, New Jersey) medium at 37°C under aerobic conditions overnight.

H. *pylori* NCTC 11637 was obtained from ATCC. Aseptically transfer *H. pylori* NCTC 11637 to a tube containing Trypticase Soy Agar/Broth (ATCC Medium 18, NCTC, 5–6 mL). Additional test tubes can be inoculated by transferring the primary broth (0.5 mL)to these secondary tubes. Use several drops from the primary broth tube to inoculate a Trypticase Soy Agar/Broth with defibrinated sheep blood (ATCC Medium 260, NCTC). Incubate at 37°C under microaerophilic conditions for 3–5 days.

### 4.3 Bacterial Culture and EV Isolation

*SA* was cultured under standard anaerobic conditions in BHI medium supplemented with appropriate growth factors. Extracellular vesicles (EVs) were isolated from bacterial culture supernatants using ultracentrifugation. Briefly, the cultures were harvested by centrifugation at 3000 × g for 30 min at 4°C to remove bacterial cells. Vesicles were subsequently concentrated by ultracentrifugation at 1 00 000 × g for 45 min at 4°C using a Beckman Culter Optima XPN‐100 with a 32Ti rotor. The resulting vesicle pellet was resuspended in phosphate‐buffered saline (PBS), and protein concentration was determined using the BCA assay. The vesicle preparations were stored at −80°C until further use.

### 4.4 Transmission Electron Microscopy (TEM) and Vesicle Size Measurement

*SA*‐EVs were visualized and characterized using transmission electron microscopy (TEM). Briefly, the isolated *SA*‐EVs were fixed with 2% paraformaldehyde and deposited onto formvar‐coated copper grids. After allowing the vesicles to settle, the grids were rinsed with phosphate‐buffered saline (PBS), followed by staining with 2% uranyl acetate for contrast enhancement. Images were captured using a TEM model operating at 80 kV.

The vesicle diameter was measured using nanoparticle tracking analysis (NTA) performed with a NTA instrument model. Diluted *SA*‐EVs samples were introduced into the analysis chamber, and the particle size distribution was recorded and analyzed using the corresponding software.

### 4.5 Western Blots

Protein samples from *SA*‐EVs were lysed in RIPA buffer containing protease inhibitors. Equal amounts of protein were separated by SDS‐PAGE and transferred onto PVDF membranes. Membranes were blocked with 5% non‐fat milk in TBST (Tris‐buffered saline with 0.1% Tween‐20) for 1 h at room temperature and incubated overnight at 4°C with primary antibodies against LTA. After washing, membranes were incubated with HRP‐conjugated secondary antibodies for 1 h at room temperature. Signals were detected using enhanced chemiluminescence (ECL) reagents and imaged using a chemiluminescent imaging system. Band intensities were quantified using ImageJ software.

### 4.6 Cellular Uptake Assay

Human gastric epithelial cells (GES‐1) were cultured in RPMI 1640 medium supplemented with 10% fetal bovine serum (FBS). *SA*‐EVs were fluorescently labeled with DEEP RED dye and incubated with gastric epithelial cells for 6 h at 37°C. Cellular uptake was analyzed by confocal microscopy and flow cytometry to confirm internalization of the EVs. Cells were washed with PBS to remove unbound EVs before imaging

### 4.7 SA‐EVs Infection in Conventional Mice

Female BALB/c mice (6–8 weeks old) were randomly divided into three groups: PBS(negative control), *H. pylori* NCTC 11637 (positive control), and *SA‐*EVs. Mice in the PBS and *SA‐*EVs groups were orally administered either broth medium (200 µL) or bacterial vesicles (20 µg) every two days. Mice in the *H. pylori* NCTC 11637 group were inoculated with *H. pylori* (2.16 × 10^9^ CFU in 200 µL BHI broth) and dosed 45 times over 3 months to ensure successful colonization of the stomach. Mice from each group were sacrificed at 2 weeks and 3 months post‐infection. All animal experiments were approved by the Animal Experimentation Ethics Committee of Southern Medical University. (Ethics: IACUC‐LAC‐20241204‐007, IACUC‐LAC‐20240315‐003).

### 4.8 Fluorescence In Situ Hybridization (FISH)

An FITC‐labeled *S. anginosus* probe (sequence: 5’‐AGT TAA ACA GTT TCC AAA GCC TAC‐3’) was used to detect *S. anginosus* colonization in paraffin‐embedded gastric sections. After deparaffinization and rehydration, the specimens were treated with HCl (0.2 N) and Proteinase K for 10 min each. Following incubation with blocking buffer at 55°C for 2 h, the FITC‐labeled *S. anginosus* probe (diluted 1:50 in 35% hybridization buffer and pre‐heated at 88°C for 3 min) was applied and hybridized in a dark, humid chamber at 42°C overnight. The specimens were then washed with a wash solution (20 mm Tris‐HCl, pH 7.2; 40 mm NaCl) and mounted using a DAPI‐antifade solution. Fluorescent images were captured with a Leica microscope.

### 4.9 Histological Evaluation

The stomachs of the mice were opened along the greater curvature and washed with cold PBS (pH 7.4). Non‐glandular regions were removed, and the remaining stomach tissue was cut into fragments, each including the body and antrum regions. Two non‐adjacent fragments (2–3 mm thick) were fixed in freshly prepared 10% formalin solution. Paraffin‐embedded gastric sections (4 µm thick) were stained with H&E and evaluated by an experienced pathologist.

### 4.10 Purified Stomach Immune Cells

The mouse gastric tissue was prepared under aseptic conditions. The tissue was then opened longitudinally and cleansed with PBS 3 times. Following that, the tissue was segmented into 0.5 cm fragments and balanced in a D‐Hanks buffer that contained 5% fetal bovine serum (FBS), HEPES (20 mm), EDTA‐Na2 (5 mm), and dithiothreitol (DTT, 2 mm). These fragments were incubated twice at 37°C for twenty‐minute intervals, agitating at 220 rpm. Following this, the gastric fragments were submitted to a 30 min incubation on ashaker at 37°C in Hanks buffer with 5% FBS, collagenase I (500 ug/mL, Beyotime, Shanghai), DNase I (50 ug/mL, Beyotime), and Dispase Il (0.5 U/mL, Thermo). The mixture was passed through a 70 um cell strainer beforebeing centrifuged at 300xg for 5 min. Immune cells from the gastric were then purifed witha density gradient centrifugation technique using a 40% and 80% gradient Percoll‐IMDM solution.

### 4.11 Flow Cytometry

Cells were first exposed to an Fc receptor‐blocking antibody (BioLegend, USA), followed by staining with specific antibodies targeting surface markers at 4°C for 30 min. For the Aldefluor assay, single‐cell suspensions were prepared according to the manufacturer's protocol using BAAA(5 µL), with or without Diethylaminobenzaldehyde (DEAB), and incubated for 30 min at 37°C. For cytokine staining, the single‐cell suspensions were treated with a protein transport inhibitor cocktail for five hours at 37°C before proceeding with the full staining process. After surface protein staining, cells were fixed using a fixation and permeabilization buffer set (Invitrogen, USA) at 4°C under light‐protected conditions for approximately 20 min. Finally, the cell suspensions were stained with antibodies against intracellular antigens and cytokines, labeled with various fluorescent dyes, and incubated under light‐protected conditions at 4°C for 30 min. The percentages of cytokine, were analyzed on a BD Canto II flow cytometer (FCM) following standard operating procedures. Data analysis was performed using BD FACSDiva software v8.02 (Becton, Dickinson and Company, USA).

### 4.12 Immunohistochemistry (IHC) Staining

Immunohistochemistry (IHC) for Ki‐67 (GB111499‐50, Servicebio), TUNEL(G1504‐50Y, Servicebio) and proliferating cell nuclear antigen (PCNA) (GB11010‐50, Servicebio) was performed on paraffin‐embedded sections (4 µm thick). The proliferation index was determined as the percentage of positive cells. For each mouse, at least five random fields spanning the corpus to antrum regions were analyzed under 100× magnification.

### 4.13 Alcian Blue Staining

Paraffin‐embedded gastric sections were deparaffinized in xylene and rehydrated through a graded alcohol series. The sections were first incubated with 3% acetic acid for 10 min and then stained with Alcian blue solution (1% Alcian blue in 3% acetic acid) for 30 min. Following a counterstain with nuclear fast red for 10 min, the slides were washed under running tap water, dehydrated through a graded ethanol series, and mounted.

### 4.14 Immunofluorescence (IF) Staining

Immunofluorescence (IF) staining for Claudin18 (GB114352‐50, Servicebio), Occludin (GB111401‐50, Servicebio), Z0‐1 (GB111981‐50, Servicebio), GIF (CSB‐PA009417YA01HU, CUSABIO TECHNOLOGY LLC) was performed on paraffin‐embedded gastric tissue sections (4 µm thick). Fluorescent images were captured using a Leica microscope.

### 4.15 LC‐MS/MS Analyze

Appropriate samples were mixed with SDT lysate (4% SDS, 100 mm Tris‐HCl) and a suitable amount of TCEP/CAA mixture. The samples were boiled at 100°C for 5 min to reduce the proteins. After cooling, UA buffer (8 m urea, 150 mm Tris‐HCl, pH 8.0) was added, mixed thoroughly, and transferred to a 10 kDa ultrafiltration centrifuge tube. The samples were centrifuged at 12 000 g for 15 min. The filter was washed twice with UA buffer (100 µL), followed by two washes with 50 mm NH_4_HCO_3_ buffer (100 µL), each time centrifuging at 12 000 g for 10 min. Trypsin buffer (20 ng/µL, 6 µg trypsin in 40 µL 50 mm NH_4_HCO_3_) was added, mixed at 600 rpm for 1 min, and incubated at 37°C for 16–18 h for digestion. The resulting peptides were collected by centrifugation at 12 000 g for 10 min. The peptides were desalted using a C18 StageTip and dried under vacuum. The dried peptides were redissolved in 0.1% formic acid, and the concentration was determined before LC‐MS analysis. Peptides were analyzed using a Q‐Exactive Plus mass spectrometer (Thermo Scientific) with data‐dependent acquisition (DDA).

### 4.16 Transcriptomic Analysis

Polyadenylated mRNA was isolated from total RNA using oligo(dT) magnetic beads. The purified RNA was then fragmented into approximately 300 bp pieces via ion disruption. This fragment size was selected to optimize sequencing efficiency—shorter fragments would increase adapter sequence representation and reduce valid data yield, while longer fragments would compromise cluster generation during sequencing. First‐strand cDNA synthesis was performed using random hexamer primers and reverse transcriptase with the fragmented RNA as template, followed by second‐strand cDNA synthesis. After library construction, PCR amplification was employed to enrich library fragments. Libraries were size‐selected for 450 bp fragments and quality‐controlled using an Agilent 2100 Bioanalyzer to assess total and effective library concentrations.

Based on the effective library concentrations and required sequencing depth, libraries containing unique index sequences were pooled in appropriate ratios (each sample was assigned a distinct index to enable bioinformatic demultiplexing). The pooled libraries were normalized to 2 nm and converted to single‐stranded DNA through alkaline denaturation. Following RNA extraction, purification, and library preparation, next‐generation sequencing (NGS) was performed using the Illumina platform with paired‐end (PE) sequencing chemistry.

### 4.17 Non‐Targeted Metabolomics Analysis

Samples were thawed at 4°C, weighed into grinding tubes, and homogenized with two steel balls in of pre‐cooled methanol‐water solution (400 µL, 4:1, v/v) using a tissue crusher at low temperature. An additional pre‐cooled methanol‐water solution (600 µL, 4:1, v/v) was added, followed by ultrasonic treatment in an ice bath for 20 min. The samples were then left to stand at ‐20°C for 1 h and centrifuged at 16 000 g for 20 min at 4°C. The supernatant was collected and dried using a high‐speed vacuum centrifuge. For mass spectrometry analysis, the dried samples were reconstituted in pre‐cooled methanol‐water (1:1, v/v) solution, centrifuged at 20 000 g for 15 min at 4°C, and the appropriate amount of supernatant was taken for analysis. Throughout the analysis, the samples were kept in a 4°C autosampler. Samples were separated using a SHIMADZU‐LC30 ultra‐high performance liquid chromatography (UHPLC) system equipped with an ACQUITY UPLC HSS T3 column (2.1 × 100 mm, 1.8 µm, Waters, Milford, MA, USA). Electrospray ionization (ESI) was used to detect both positive (+) and negative (−) ion modes. Following UPLC separation, samples were analyzed using a QE Plus mass spectrometer (Thermo Scientific).

### 4.18 16S rRNA Sequencing

Total DNA was extracted from microbiome samples using optimized methods. DNA quality was verified by Nanodrop quantification and 0.8% agarose gel electrophoresis. For multiplexed sequencing, we performed two‐step PCR with: First round: PacBio universal sequence + target‐specific primers (16S/ITS/archaeal). Second round: Sample‐specific barcode + PacBio universal sequence. PCR used Q5 High‐Fidelity Polymerase (NEB) with minimized cycles. Negative controls monitored contamination. Products were size‐selected (2% gel), purified (AXYGEN kit), and quantified (PicoGreen/BioTek reader). Equimolar pools were prepared for PacBio sequencing using Template Prep Kit 1.0.

### 4.19 Serum Alanine Transaminase (ALT), Aspartate Aminotransferas (AST) and Albumin(ALB) Detection

Serum levels of ALT, AST, and ALB were assessed using a Chemistry Analyzer following the manufacturer's instructions. Fifty microliters of serum were diluted to 200 µL with PBS, and the diluted serum, along with slides for ALT, AST, and ALB, were loaded simultaneously for automatic analysis.

### 4.20 Construction of S. Anginosus △Tmpc and △Fbp62 Strain

The knockout procedure was followed in previous studies [5, 13]. The upstream and downstream flanking regions of the *Tmpc* and *Fbp62* genes were synthesized using chromosomal DNA from *Streptococcus anginosus* ATCC 33397 as the template (NCBI Accession Number: NARD01000001; retrieved from https://www.ncbi.nlm.nih.gov/datasets/genome/GCF_002088025.1/). The scheme map of homology arms for recombinant is illustrated in Figure 10. The ermB gene was amplified from plasmid pGL0_160 [ermB] (Addgene Plasmid #210682), while the *tetR* gene was amplified from plasmid pRF‐TetR (Addgene Plasmid #49374). The *ermB* gene was inserted into the *Tmpc* gene, and the resulting mutant Tmpc gene was cloned into plasmid pUC19 to generate the recombinant plasmid pUC19‐*Tmpc*△*ermB*. Moreover, the *tetR* gene was integrated into plasmid pUC57 and inserted into the *Fbp62* gene locus to obtain the recombinant plasmid pUC57‐*Fbp62*△*tetR*. *Streptococcus anginosus* cells (OD600 = 0.02) were incubated with competence‐stimulating peptide 1 (CSP‐1, 50 µg/mL) and the aforementioned recombinant plasmid DNA (1 µg/mL) at 37°C for 3 h, followed by plating on THY agar supplemented with erythromycin or tetracycline. Colonies were selected after 48 h of incubation and screened for successful gene insertion by PCR. Bacterial chromosomal DNA was extracted using the QIAamp DNA Mini Kit (Qiagen, Cat. No. 51304) and amplified with primer pairs *Tmpc*‐F/R or *Fbp62*‐F/R to verify the insertion of the *ermB* and *tetR* genes. After screening, the mutant *Tmpc*, *Fbp62* single mutant or *Tmpc* plus *Fbp62* mutant *SA* were applied for further experiments to cultured and isolated the extracellular vesicles. The primers for amplified the *Tmpc*: *Tmpc*‐FWD‐P1: 5’‐GAACAAGAAACAATGGCTAGGC‐3’ and *Tmpc*‐REV‐P2: 5’‐CGTCAAGGATTTTTGCTTTTGC‐3’; while the *Fbp62* primers were: *Fbp62*‐FWD‐P1: 5’‐CACCACATGACAGAGGAATTGC‐3’ and *Fbp62*‐REV‐P1: 5’‐TTGGCTTCATCGGGAGTGACC‐3’.

### 4.21 DNA Extraction and Electrophoresis

Stomach tissue samples were collected from mice and subjected to DNA extraction using a DNA lysis buffer (9170A, TaKaRa) following standard protocols. The extracted DNA was then amplified using polymerase chain reaction (PCR), and the resulting amplified products were analyzed by agarose gel electrophoresis to assess DNA integrity and confirm target amplification.

### 4.22 Purification of TMPC and FBP62

As previously established [27], the Tmpc and Fbp62 genes were amplified via polymerase chain reaction (PCR) utilizing primers incorporating NdeI and XhoI restriction enzyme cloning sites. Subsequently, the amplified genes were cloned into the prokaryotic expression vector pET‐30a (+) and introduced into *Escherichia coli (E. coli)* strain BL21(DE3). E. coli transformants harboring plasmids encoding His‐tagged TMPC and FBP62 fusion proteins were cultured in lysogeny broth (LB) medium supplemented with 0.4 mm isopropyl‐β‐D‐thiogalactopyranoside (IPTG) to induce the expression of target proteins. The expression efficiency of the recombinant fusion proteins was initially assessed via Coomassie Brilliant Blue staining. Following a 12 h incubation at 25°C, E. coli cells were harvested and lysed in lysis buffer [20 mm Tris–HCl (pH 8.0), 150 mm NaCl], prior to subsequent disruption using a high‐pressure cell disruptor (Union‐Biotech Co., Ltd.). The resulting cell lysate supernatants were collected and loaded onto a Ni‐NTA agarose resin column (Qiagen), followed by washing with a buffer containing Tris‐HCl (20 mm, pH 8.0), NaCl (150 mm), and imidazole (20 mm) to remove non‐specifically bound proteins. The target proteins were further purified via HiTrap ion exchange chromatography and Superdex 200 Increase 10/300 GL gel filtration chromatography (GE Healthcare Life Sciences). The purity and identity of the purified TMPC and FBP62 proteins were validated through Western blot analysis using a His‐tag‐specific polyclonal antibody.

### 4.23 Preparation of Antibodies Targeting TMPC and FBP62

Twelve‐week‐old male New Zealand White rabbits were immunized with purified TMPC and FBP62 proteins emulsified in Freund's Complete Adjuvant (FCA). Thereafter, the rabbits received weekly booster immunizations with the same TMPC and FBP62 proteins emulsified in Freund's Incomplete Adjuvant (FIA) for a duration of 3 weeks. Rabbit antisera were collected 10 days following the final booster injection, in accordance with previously established protocols. The specificity of the generated anti‐TMPC and anti‐FBP62 antibodies was evaluated by detecting the respective target proteins in three experimental groups: *E. coli* BL21(DE3) (serving as a Gram‐negative bacterial negative control), Tmpc‐deficient and Fbp62‐deficient *Streptococcus anginosus (SA)* mutant strains, and wild‐type *SA*.

### 4.24 Statistical Analysis

Data is shown as mean ± SD, with dots representing individual donors (average of technical duplicates, n = 6). Statistical differences between groups were determined using one‐way ANOVA with Tukey post‐tests. *p* < 0.05 (*); *p* < 0.01 (**); *p* < 0.001 (***); *p* < 0.0001 (****); ns: not significant. Statistical analyses were performed using GraphPad Prism 9 (Graphpad Software, San Diego, CA). Specific statistical tests are annotated within the respective figure legends.

## Author Contributions

Y.G., L.N.D., and H.X.L. designed the study, performed experiments, and wrote the manuscript. J.X., and Y.L.D. performed experiments, analyzed the data, and wrote the manuscript. H.X.W., Y.J.Z., X.M.H., and H.F.W. performed experiments, and analyzed the data. T.X.A., X.L., Y.R.Q., and L.Z. participated in the discussion of data and revised the manuscript. Y.G., L.Z., and H.X.L. supervised the study and took responsibility for the contents as guarantors of this article. All the authors reviewed and approved the manuscript.

## Funding

This work was financially supported by grant from the National Key R&D Program of China on Cancer, Cardiovascular and Cerebrovascular, Respiratory, and Metabolic Diseases (No. 2025ZD0543900), the National Natural Science Foundation of China (82002218, 82202978), Guangdong Basic and Applied Basic Research Foundation (2021A1515110821), Guangzhou Basic and Applied Basic Research Foundation (2023A04J2359), outstanding Youths Development Scheme of Nanfang Hospital, Southern Medical University (2024J007), Project funded by the Wu Jieping Medical Foundation(320.6750.2025‐6‐104), Guangdong Provincial Clinical Research Center for Laboratory Medicine (2023B110008), and Ganzhou “Technology + Healthcare” Joint Program Project (Key Project) Fund (2025YLCE0012).

## Ethics Statement

This study was approved by the Ethics Committee of Nanfang Hospital, Southern Medical University (IACUC‐LAC‐20241204‐007, IACUC‐LAC‐20240315‐003).

## Conflicts of Interest

The authors declare no conflicts of interest.

## Supporting information




**Supporting File 1**: advs74089‐sup‐0001‐SuppMat.docx.

**Supporting File 2**: advs74089‐sup‐0002‐Figure S1‐S9.pdf.

**Supporting File 3**: advs74089‐sup‐0003‐Table S1.xlsx.

**Supporting File 4**: advs74089‐sup‐0004‐Table S2.xlsx.

**Supporting File 5**: advs74089‐sup‐0005‐Table S3.xlsx.

**Supporting File 6**: advs74089‐sup‐0006‐Table S4.xlsx.

**Supporting File 7**: advs74089‐sup‐0007‐Table S5.xlsx.

**Supporting File 8**: advs74089‐sup‐0008‐Table S6.xlsx.

**Supporting File 9**: advs74089‐sup‐0009‐Table S7.xlsx.

**Supporting File 10**: advs74089‐sup‐0010‐DataFile.xlsx.

## Data Availability

The data that support the findings of this study are available in the supplementary material of this article.

## References

[1] X. Gao , P. Yin , Y. Ren , et al., “Predicting Personalized Diets Based on Microbial Characteristics Between Patients With Superficial Gastritis and Atrophic Gastritis,” Nutrients 15 (2023): 4738, 10.3390/nu15224738. 38004131 PMC10675729

[2] E. Tshibangu‐Kabamba and Y. Yamaoka , “Helicobacter Pylori Infection and Antibiotic Resistance — From Biology to Clinical Implications,” Nature Reviews Gastroenterology & Hepatology 18 (2021): 613–629, 10.1038/s41575-021-00449-x. 34002081

[3] J. Fan , J. Zhu , and H. Xu , “Strategies of Helicobacter Pylori in Evading Host Innate and Adaptive Immunity: Insights and Prospects for Therapeutic Targeting,” Frontiers in Cellular and Infection Microbiology 14 (2024): 1342913, 10.3389/fcimb.2024.1342913. 38469348 PMC10925771

[4] P. Malfertheiner , M. C. Camargo , E. El‐Omar , et al., “Helicobacter Pylori Infection,” Nature Reviews Disease Primers 9 (2023): 19, 10.1038/s41572-023-00431-8. PMC11558793 37081005

[5] K. Fu , A. H. K. Cheung , C. C. Wong , et al., “Streptococcus Anginosus Promotes Gastric Inflammation, Atrophy, and Tumorigenesis in Mice,” Cell 187 (2024): 882–896, 10.1016/j.cell.2024.01.004. 38295787

[6] C.‐B. Zhou , S.‐Y. Pan , P. Jin , et al., “Fecal Signatures of Streptococcus Anginosus and Streptococcus Constellatus for Noninvasive Screening and Early Warning of Gastric Cancer,” Gastroenterology 162 (2022): 1933–1947, 10.1053/j.gastro.2022.02.015. 35167866

[7] O. O. Coker , Z. Dai , Y. Nie , et al., “Mucosal Microbiome Dysbiosis in Gastric Carcinogenesis,” Gut 67 (2018): 1024–1032, 10.1136/gutjnl-2017-314281. 28765474 PMC5969346

[8] X. Xiao , X. Zhang , J. Wang , et al., “Proton Pump Inhibitors Alter Gut Microbiota by Promoting Oral Microbiota Translocation: A Prospective Interventional Study,” Gut 73 (2024): 1098–1109, 10.1136/gutjnl-2023-330883. 38267200

[9] M. Pilarczyk‐Zurek , J. Budziaszek , K. Nandagopal , et al., “Streptococcus Anginosus Orchestrates Antibacterial Potential of NETs Facilitating Survival of Accompanying Pathogens,” Microbiological Research 290 (2025): 127959, 10.1016/j.micres.2024.127959. 39489135

[10] B. Y. Sunwoo and W. T. Miller Jr. , “Streptococcus Anginosus Infections,” Chest 146 (2014): e121–e125, 10.1378/chest.13-2791. 25288003

[11] M. Hui , “Streptococcus Anginosus Bacteremia: Sutton's Law,” Journal of Clinical Microbiology 43 (2005): 6217, 10.1128/jcm.43.12.6217.2005. 16333137 PMC1317220

[12] A. Kuryłek , M. Stasiak , and I. Kern‐Zdanowicz , “Virulence Factors of Streptococcus Anginosus—A Molecular Perspective,” Frontiers in Microbiology 13 (2022): 1025136, 10.3389/fmicb.2022.1025136. 36386673 PMC9643698

[13] Y. Kodama , T. Ishikawa , Y. Shimoyama , D. Sasaki , S. Kimura , and M. Sasaki , “The Fibronectin‐Binding Protein Homologue Fbp62 of Streptococcus Anginosus is a Potent Virulence Factor,” Microbiology and Immunology 62 (2018): 624–634, 10.1111/1348-0421.12646. 30192020

[14] Q. Gao , W. Zhou , N. Nurxat , et al., “Dynamic Profiling of Lipoteichoic Acid (LTA) and/or Lipopolysaccharide (LPS) Positive Extracellular Vesicles in Plasma as Diagnostic and Prognostic Biomarkers for Bacterial Infection,” Advanced Science 12 (2025): 06613, 10.1002/advs.202506613. PMC12667508 40903799

[15] X. Liang , N. Dai , K. Sheng , et al., “Gut Bacterial Extracellular Vesicles: Important Players in Regulating Intestinal Microenvironment,” Gut Microbes 14 (2022): 2134689, 10.1080/19490976.2022.2134689. 36242585 PMC9578468

[16] L. Duan , W. Lin , Y. Zhang , et al., “Exosomes in Autoimmune Diseases: A Review of Mechanisms and Diagnostic Applications,” Clinical Reviews in Allergy & Immunology 68 (2025): 5, 10.1007/s12016-024-09013-2. 39820756

[17] J. A. Welsh , D. C. Goberdhan , L. O'Driscoll , et al., “Minimal Information for Studies of Extracellular Vesicles (MISEV2023): From Basic to Advanced Approaches,” Journal of Extracellular Vesicles 13 (2024): 12404, 10.1002/jev2.12404. PMC10850029 38326288

[18] W. Huang , L. Meng , Y. Chen , Z. Dong , and Q. Peng , “Bacterial Outer Membrane Vesicles as Potential Biological Nanomaterials for Antibacterial Therapy,” Acta Biomaterialia 140 (2022): 102–115, 10.1016/j.actbio.2021.12.005. 34896632

[19] H. S. Han , S. Hwang , S. Y. Choi , et al., “Roseburia Intestinalis ‐Derived Extracellular Vesicles Ameliorate Colitis by Modulating Intestinal Barrier, Microbiome, and Inflammatory Responses,” Journal of Extracellular Vesicles 13 (2024): 12487, 10.1002/jev2.12487. PMC11336657 39166405

[20] N. Díaz‐Garrido , J. Badia , and L. Baldomà , “Microbiota‐Derived Extracellular Vesicles in Interkingdom Communication in the Gut,” Journal of Extracellular Vesicles 10 (2021): 12161, 10.1002/jev2.12161. PMC8568775 34738337

[21] R. Kalluri and V. S. LeBleu , “The Biology, Function, and Biomedical Applications of Exosomes,” Science 367 (2020): aau6977, 10.1126/science.aau6977. PMC7717626 32029601

[22] M. Lu , W. Shao , H. Xing , and Y. Huang , “Extracellular Vesicle‐Based Nucleic Acid Delivery,” Interdisciplinary Medicine 1 (2023): 20220007, 10.1002/inmd.20220007.

[23] Q. Li , B. Li , Z. Chen , et al., “Requirements for Human Pluripotent Stem Cell Derived Small Extracellular Vesicles,” Interdisciplinary Medicine 1 (2023): 20220018, 10.1002/inmd.20220018.

[24] A. Chronopoulos and R. Kalluri , “Emerging Role of Bacterial Extracellular Vesicles in Cancer,” Oncogene 39 (2020): 6951–6960, 10.1038/s41388-020-01509-3. 33060855 PMC7557313

[25] L. Brown , J. M. Wolf , R. Prados‐Rosales , and A. Casadevall , “Through the Wall: Extracellular Vesicles in Gram‐Positive Bacteria, Mycobacteria and Fungi,” Nature Reviews Microbiology 13 (2015): 620–630, 10.1038/nrmicro3480. 26324094 PMC4860279

[26] H.‐I. Choi , J.‐P. Choi , J. Seo , et al., “Helicobacter Pylori‐Derived Extracellular Vesicles Increased in the Gastric Juices of Gastric Adenocarcinoma Patients and Induced Inflammation Mainly via Specific Targeting of Gastric Epithelial Cells,” Experimental & Molecular Medicine 49 (2017): 330, 10.1038/emm.2017.47. PMC5454444 28496197

[27] M. Hong , Z. Li , H. Liu , et al., “Fusobacterium Nucleatum Aggravates Rheumatoid Arthritis Through FadA‐Containing Outer Membrane Vesicles,” Cell Host & Microbe 31 (2023): 798–810, 10.1016/j.chom.2023.03.018. 37054714

[28] H. Kayama , R. Okumura , and K. Takeda , “Interaction Between the Microbiota, Epithelia, and Immune Cells in the Intestine,” Annual Review of Immunology 38 (2020): 23–48, 10.1146/annurev-immunol-070119-115104. 32340570

[29] M. Wen , J. Wang , Z. Ou , et al., “Bacterial Extracellular Vesicles: A Position Paper by the Microbial Vesicles Task Force of the Chinese Society for Extracellular Vesicles,” Interdisciplinary Medicine 1 (2023): 20230017, 10.1002/INMD.20230017.

[30] Y. Chen , Z. Ou , M. Pang , et al., “Extracellular Vesicles Derived From Akkermansia Muciniphila Promote Placentation and Mitigate Preeclampsia in a Mouse Model,” Journal of Extracellular Vesicles 12 (2023): 12328, 10.1002/jev2.12328. 37165987 PMC10173384

[31] J. Ghanam , V. K. Chetty , L. Barthel , D. Reinhardt , P.‐F. Hoyer , and B. K. Thakur , “DNA in Extracellular Vesicles: From Evolution to Its Current Application in Health and Disease,” Cell & Bioscience 12 (2022): 37, 10.1186/s13578-022-00771-0. 35346363 PMC8961894

[32] S. Liu , L. Hong , S. Zhang , et al., “Sporisorium Reilianum Polysaccharides Improve DSS‐Induced Ulcerative Colitis by Regulating Intestinal Barrier Function and Metabolites,” International Journal of Biological Macromolecules 265 (2024): 130863, 10.1016/j.ijbiomac.2024.130863. 38490380

[33] Y.‐F. Wei , X. Li , M.‐R. Zhao , et al., “Helicobacter Pylori Disrupts Gastric Mucosal Homeostasis by Stimulating Macrophages to Secrete CCL3,” Cell Communication and Signaling 22 (2024): 263, 10.1186/s12964-024-01627-5. 38730482 PMC11084090

[34] Z. Chen , Z. Tang , W. Li , et al., “Weizmannia Coagulans BCF‐01: A Novel Gastrogenic Probiotic for Helicobacter Pylori Infection Control,” Gut Microbes 16 (2024): 2313770, 10.1080/19490976.2024.2313770. 38334087 PMC10860349

[35] N. Ghosh , K. Kesh , P. K. Singh , et al., “Morphine Use Induces Gastric Microbial Dysbiosis Driving Gastric Inflammation Through TLR2 Signalling Which Is Attenuated by Proton Pump Inhibition,” British Journal of Pharmacology 180 (2023): 1582–1596, 10.1111/bph.16025. 36585367 PMC10175111

[36] J. K. Dowling , R. Afzal , L. J. Gearing , et al., “Mitochondrial Arginase‐2 Is Essential for IL‐10 Metabolic Reprogramming of Inflammatory Macrophages,” Nature Communications 12 (2021): 1460, 10.1038/s41467-021-21617-2. PMC7936006 33674584

[37] C. Morse , T. Tabib , J. Sembrat , et al., “Proliferating SPP1/MERTK‐Expressing Macrophages in Idiopathic Pulmonary Fibrosis,” European Respiratory Journal 54 (2019): 1802441, 10.1183/13993003.02441-2018. 31221805 PMC8025672

[38] W. Yoo , N. G. Shealy , J. K. Zieba , et al., “Salmonella Typhimurium Expansion in the Inflamed Murine Gut Is Dependent on Aspartate Derived From ROS‐Mediated Microbiota Lysis,” Cell Host & Microbe 32 (2024): 887–899, 10.1016/j.chom.2024.05.001. 38806059 PMC11189616

[39] A. S. Krall , P. J. Mullen , F. Surjono , et al., “Asparagine Couples Mitochondrial Respiration to ATF4 Activity and Tumor Growth,” Cell Metabolism 33 (2021): 1013–1026, 10.1016/j.cmet.2021.02.001. 33609439 PMC8102379

[40] Z. Liu , D. Zhang , and S. Chen , “Unveiling the Gastric Microbiota: Implications for Gastric Carcinogenesis, Immune Responses, and Clinical Prospects,” Journal of Experimental & Clinical Cancer Research 43 (2024): 118, 10.1186/s13046-024-03034-7. 38641815 PMC11027554

[41] I. Nakayama , C. Qi , Y. Chen , Y. Nakamura , L. Shen , and K. Shitara , “Claudin 18.2 as a Novel Therapeutic Target,” Nature Reviews Clinical Oncology 21 (2024): 354–369, 10.1038/s41571-024-00874-2. 38503878

[42] A. K. Srivastava , B. S. Venkata , Y. Y. Sweat , et al., “Serine 408 Phosphorylation Is a Molecular Switch That Regulates Structure and Function of the Occludin α‐helical Bundle,” Proceedings of the National Academy of Sciences 119 (2022): 2204618119, 10.1073/pnas.2204618119. PMC9407527 35969745

[43] W.‐T. Kuo , L. Zuo , M. A. Odenwald , et al., “The Tight Junction Protein ZO‐1 Is Dispensable for Barrier Function but Critical for Effective Mucosal Repair,” Gastroenterology 161 (2021): 1924–1939, 10.1053/j.gastro.2021.08.047. 34478742 PMC8605999

[44] E. Palacios , L. Lobos‐González , S. Guerrero , et al., “Helicobacter Pylori Outer Membrane Vesicles Induce Astrocyte Reactivity Through Nuclear Factor‐κappa B Activation and Cause Neuronal Damage in Vivo in a Murine Model,” Journal of Neuroinflammation 20 (2023): 66, 10.1186/s12974-023-02728-7. 36895046 PMC9996972

[45] M. Chmiela , N. Walczak , and K. Rudnicka , “Helicobacter Pylori Outer Membrane Vesicles Involvement in the Infection Development and Helicobacter Pylori‐Related Diseases,” Journal of Biomedical Science 25 (2018): 78, 10.1186/s12929-018-0480-y. 30409143 PMC6225681

[46] D. C. Fajgenbaum and C. H. June , “Cytokine Storm,” New England Journal of Medicine 383 (2020): 2255–2273, 10.1056/NEJMra2026131. 33264547 PMC7727315

[47] C. Liu , D. Chu , K. Kalantar‐Zadeh , J. George , H. A. Young , and G. Liu , “Cytokines: From Clinical Significance to Quantification,” Advanced Science 8 (2021): 2004433, 10.1002/advs.202004433. 34114369 PMC8336501

[48] S. K. Heuser , J. Li , S. Pudewell , A. LoBue , Z. Li , and M. M. Cortese‐Krott , “Biochemistry, Pharmacology, and In Vivo Function of Arginases,” Pharmacological Reviews 77 (2025): 100015, 10.1124/pharmrev.124.001271. 39952693 PMC12105760

[49] M. O. Johnson , M. M. Wolf , M. Z. Madden , et al., “Distinct Regulation of Th17 and Th1 Cell Differentiation by Glutaminase‐Dependent Metabolism,” Cell 175 (2018): 1780–1795, 10.1016/j.cell.2018.10.001. 30392958 PMC6361668

[50] T. Xu , K. M. Stewart , X. Wang , et al., “Metabolic Control of TH17 and Induced Treg Cell Balance by an Epigenetic Mechanism,” Nature 548 (2017): 228–233, 10.1038/nature23475. 28783731 PMC6701955

[51] J. K. Nicholson , E. Holmes , J. Kinross , et al., “Host‐Gut Microbiota Metabolic Interactions,” Science 336 (2012): 1262–1267, 10.1126/science.1223813. 22674330

[52] T. Hu , C.‐H. Liu , M. Lei , et al., “Metabolic Regulation of the Immune System in Health and Diseases: Mechanisms and Interventions,” Signal Transduction and Targeted Therapy 9 (2024): 268, 10.1038/s41392-024-01954-6. 39379377 PMC11461632

[53] C. H. Patel , R. D. Leone , M. R. Horton , and J. D. Powell , “Targeting Metabolism to Regulate Immune Responses in Autoimmunity and Cancer,” Nature Reviews Drug Discovery 18 (2019): 669–688, 10.1038/s41573-019-0032-5. 31363227

[54] L. Jin , J. Xiao , Y. Luo , et al., “Exploring Gut Microbiota in Systemic Lupus Erythematosus: Insights and Biomarker Discovery Potential,” Clinical Reviews in Allergy & Immunology 68 (2025): 42, 10.1007/s12016-025-09051-4. 40216660

[55] J. Xiao , Y. Luo , L. Duan , et al., “Exploring Differential Gene Expression and Biomarker Potential in Systemic Lupus Erythematosus: A Retrospective Study,” PeerJ 13 (2025): 19891, 10.7717/peerj.19891. PMC12424612 40949741

[56] B. H. Min , S. Devi , G. H. Kwon , et al., “Gut Microbiota‐Derived Indole Compounds Attenuate Metabolic Dysfunction‐Associated Steatotic Liver Disease by Improving Fat Metabolism and Inflammation,” Gut Microbes 16 (2024): 2307568, 10.1080/19490976.2024.2307568. 38299316 PMC10841017

[57] Y. Lu , A. Cui , and X. Zhang , “Commensal Microbiota‐Derived Metabolite Agmatine Triggers Inflammation to Promote Colorectal Tumorigenesis,” Gut Microbes 16 (2024): 2348441, 10.1080/19490976.2024.2348441. 38706224 PMC11086030

[58] H. Liu , R. Huang , B. Shen , et al., “Live Akkermansia Muciniphila Boosts Dendritic Cell Retinoic Acid Synthesis to Modulate IL‐22 Activity and Mitigate Colitis in Mice,” Microbiome 12 (2024): 275, 10.1186/s40168-024-01995-7. 39734222 PMC11684322

