# Correction to “Extracellular Vesicles of *Streptococcus anginosus* Mediate Gastritis via Epithelial Barrier Disruption and Macrophage‐driven Inflammation”

**DOI:** 10.1002/advs.76534

**Published:** 2026-08-06

Ying Gong, Lina Duan, Jie Xiao, Yulu Deng, Hongxia Wang, Yajie Zhang, Xiumei Hu, Haifang Wang, Taixue An, Xin Li, Yurong Qiu, Lei Zheng, Haixia Li, “Extracellular Vesicles of *Streptococcus anginosus* Mediate Gastritis via Epithelial Barrier Disruption and Macrophage‐driven Inflammation.” *Advanced Science* 13. no. 19 (2026): e12494. https://doi.org/10.1002/advs.202512494.

The image in Figure 2B for the *E.coli*‐EVs group was misplaced due to carelessness, resulting in a duplication with the PBS group image in Figure 3B. Please find the picture with the correct photo below. The corresponding raw data Excel file has also been updated accordingly with the correct image. In addition, the statistical data for ZO‐1 in Figure 5C within the raw data file were inadvertently taken from the OCCLUDIN dataset. Although the correct data have always been accurately presented in the published figure, this error has now been rectified in the updated raw data file.


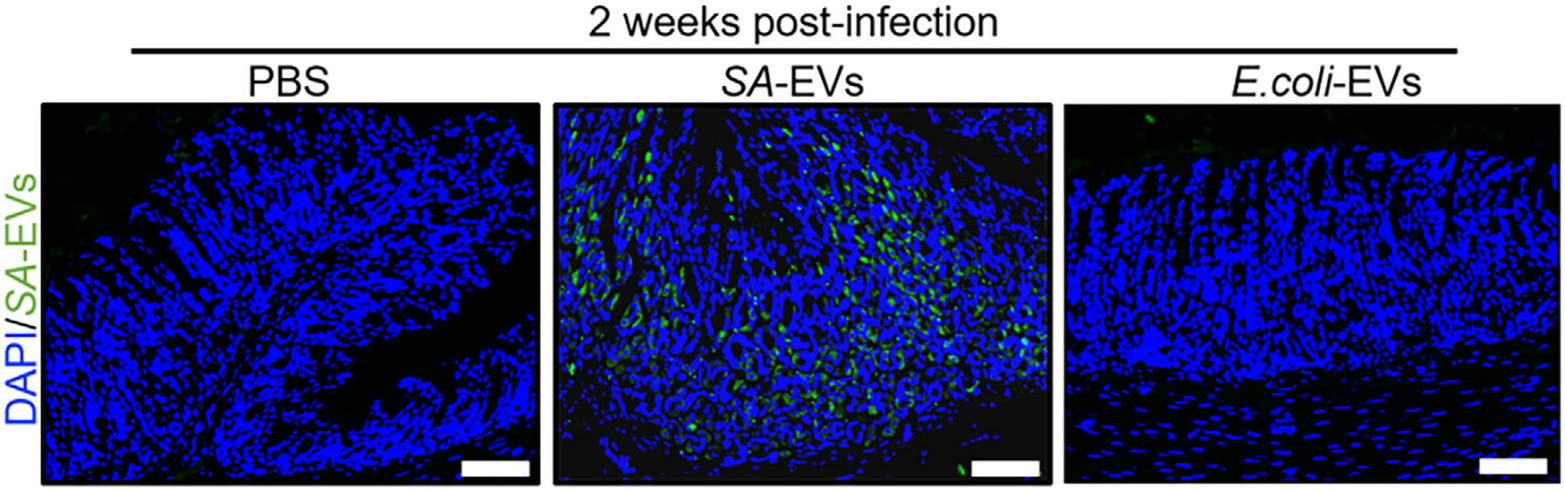


We apologize for this error.

## Supporting information




**Supporting File**: advs76534‐sup‐0001‐datafile.xlsx.

