# Supplementary material for: Extracellular Vesicles of Streptococcus anginosus Mediate Gastritis via Epithelial Barrier Disruption and Macrophage‐driven Inflammation
Source: Adv Sci (Weinh). 2026 Jan 30;13(19):e12494. doi: 10.1002/advs.202512494 (PMC13045418; doi:10.1002/advs.202512494)
Supplement: Supplementary file 1 — Supporting File 1: advs74089‐sup‐0001‐SuppMat.docx. [file ADVS-13-e12494-s009.docx]

# Extracellular vesicles of Streptococcus anginosus mediate gastritis via epithelial barrier disruption and macrophage-driven inflammation

*Ying Gong^1,2,3#^*, Lina Duan^1,2#^, Jie Xiao^1,2#^, Yulu Deng^1,2#^, Hongxia Wang^1,2^, Yajie Zhang^1,2^, Xiumei Hu^1,2^, Haifang Wang^1,2^, Taixue An^1,2^, Xin Li^1,2^, Yurong Qiu^1,2^, Lei Zheng^1,2,4^*, Haixia Li^1,2^*.*

^1.^Department of Laboratory Medicine, Guangdong Provincial Key Laboratory of Precision Medical Diagnostics, Guangdong Engineering and Technology Research Center for Rapid Diagnostic Biosensors, Guangdong Provincial Key Laboratory of Single-cell and Extracellular Vesicles, Nanfang Hospital, Southern Medical University, Guangzhou, 510515, P. R. China.

^2.^Guangdong Provincial Clinical Research Center for Laboratory Medicine.

^3.^Department of Internal Medicine, Division of Hematology, University of Maastricht, Maastricht 6229ER, Netherlands.

^4.^State Key Laboratory of Multi-organ Injury Prevention and Treatment, Nanfang Hospital, Southern Medical University, Guangzhou, China.

***Correspondence author:**

Prof. Dr. Haixia Li (Leader Contact)

Email: yingchun1220@163.com

Dr. Ying Gong

Email: [gongy3@mail2.sysu.edu.cn](mailto:gongy3@mail2.sysu.edu.cn)

Prof. Dr. Lei Zheng

Email: [nfyyzhenglei@smu.edu.cn](mailto:nfyyzhenglei@smu.edu.cn)

Funding: This work was financially supported by grant from the National Key R&D Program of China on Cancer, Cardiovascular and Cerebrovascular, Respiratory, and Metabolic Diseases (No. 2025ZD0543900), the National Natural Science Foundation of China (82002218, 82202978), Guangdong Basic and Applied Basic Research Foundation (2021A1515110821), Guangzhou Basic and Applied Basic Research Foundation (2023A04J2359), outstanding Youths Development Scheme of Nanfang Hospital, Southern Medical University (2024J007), Project funded by the Wu Jieping Medical Foundation(320.6750.2025-6-104), Guangdong Provincial Clinical Research Center for Laboratory Medicine (2023B110008) and Ganzhou "Technology + Healthcare" Joint Program Project (Key Project) Fund (2025YLCE0012).

^#^ Ying Gong, Lina Duan, Jie Xiao and Yulu Deng contributed equally to this work.

**Figure** **S1 The effect of *SA*-EVs on serum inflammatory factors in mice, related to Figure 3.**

1. ELISA analysis of Ccl8, IL-6, IL-10, and IL-22 in gastric tissue following 3 months of intragastric administration. Data is shown as mean ± SD, with dots representing individual donors (average of technical duplicates, n=6). Statistical differences between groups were determined using one-way ANOVA with Tukey post-tests. p < 0.05 (*); p < 0.01 (**); p < 0.001 (***); p < 0.0001 (****); ns: not significant.

**Figure** **S2 Effect of *SA*-EVs on gastric apoptosis, related to Figure 4 and Figure 5.**

1. Expression of Terminal deoxynucleotidyl transferase dUTP Nick-End Labeling (TUNEL) in mice infected with *SA*-EVs. Scale bars = 50 µm. Data is shown as mean ± SD, with dots representing individual donors (average of technical duplicates, n=6). Statistical differences between groups were determined using one-way ANOVA with Tukey post-tests. p < 0.05 (*); p < 0.01 (**); p < 0.001 (***); p < 0.0001 (****); ns: not significant.

**Figure** **S3 *SA*-EVs has no side effects upon long-term infection in mice, related to Figure 2 and Figure 3.**

1. (A-F)Body weight (A), daily food intake (B), Representative images of colon (C), colon length (D), Liver function index (E), and liver-to-body weight ratio (F) of *SA*-EVs and *H. pylori*-infected mice compared to PBS control. (G) PCR detection of *SA* DNA in mouse stomachs 2 weeks after oral gavage with *SA*-EVs, PBS, or DSS. n = 6 for each group. (H) PCR detection of *SA* DNA in mouse stomachs 2 weeks after oral gavage with *SA*-EVs. n = 6 for each group. (I) PCR detection of *SA* DNA in mouse stomachs 2 weeks and 3 months after oral gavage with *SA*-EVs. n = 6 for each group. (J) *SA* colonization and bacteria loads were confirmed by bacterial culture of fresh gastric mucosa tissue from *SA*-EVs-infected mice. Data is shown as mean ± SD, with dots representing individual donors (average of technical duplicates, n=6). Statistical differences between groups were determined using one-way ANOVA with Tukey post-tests. p < 0.05 (*); p < 0.01 (**); p < 0.001 (***); p < 0.0001 (****); ns: not significant.

**Figure** **S4 *SA*-EVs activates inflammation-related pathways in mice, related to Figure 7.**

(A-B) Bubble charts (A) and KEGG pathway enrichment bar charts (B) present the expression status of pathways.

**Figure** **S5 Effects of *SA*-EVs on metabolism, related to Figure 8.**

(A-C) KEGG Level 2 Pathway Enrichment Bubble Plot (A), Circular Pathway Diagram(B), and KEGG-Based Differential Metabolite Regulation Network(C) display differentially expressed proteins and associated pathways among mice with gavage of PBS or *SA*-EVs (n = 3).

**Figure** **S6 Aspartate affects metabolic pathways and regulates inflammatory responses, related to Figure 7 and figure 8.**

1. B) A significant KEGG enrichment bubble chart (A), and the p-value heatmap (B) integrate gastric transcriptomic and fecal metabolomic data from mice, revealing notable correlations. (C) The significant correlation hierarchical clustering heatmap. (D) The pathway maps of differential metabolites and proteins (genes) highlight key enzymes associated with aspartate metabolism. (E-G) The relative mRNA expression of *Agxt*, *Cps1*, and *Gls2*. Data is shown as mean ± SD, with dots representing individual donors (average of technical duplicates, n=6). Statistical differences between groups were determined using one-way ANOVA with Tukey post-tests. p < 0.05 (*); p < 0.01 (**); p < 0.001 (***); p < 0.0001 (****); ns: not significant.

**Figure** **S7 *SA*-EVs induce polarization of macrophages, related to Figure 7**

1. *SA* and *SA*-EVs were separately inoculated onto MH agar plates and blood agar plates. (B)Fecal samples were collected from mice that received PBS or *SA*-EVs via intragastric administration for two weeks or three months, and *SA* DNA was analyzed by electrophoresis. (C-E) Flow cytometric analysis of gastric tissues was performed, and macrophage expression was presented using statistical graphs, histograms, and scatter plots. Data is shown as mean ± SD, with dots representing individual donors (average of technical duplicates, n=6). Statistical differences between groups were determined using one-way ANOVA with Tukey post-tests. p < 0.05 (*); p < 0.01 (**); p < 0.001 (***); p < 0.0001 (****); ns: not significant.

**Figure** **S8 Wild-type strains and gene fragments of TMPC and FBP62 following complete gene knockout, related to Figure 10.**

(A) *Tmpc* gene fragment from the wild-type strain. (B) *Tmpc* gene fragment following gene knockout.(C) *Fbp62* gene fragment from the wild-type strain. *Fbp62* gene fragment following gene knockout.

**Figure** **S9** The TEM image of *SA* with △*Tmpc* and △*Fbp62* mutant. Scale bars = 50 µm.

**Methods**

**Transcriptome analysis flow**

1. **Quality control**

Samples are sequenced on the platform to get image files, which are transformed by the software of the sequencing platform, and the original data in FASTQ format (Raw Data) is generated. Sequencing data contains a number of connectors, low-quality Reads, so we use Cutadapt (v1.15) software to filter the sequencing data to get high quality sequence（Clean Data）for further analysis.

1. **Reads mapping**

The reference genome and gene annotation files were downloaded from genome website. The filtered reads were mapping to the reference genome using HISAT2 v2.0.5.

1. **Differential expression analysis**

we used HTSeq(0.9.1) statistics to compare the Read Count values on each gene as the original expression of the gene, and then used FPKM to standardize the expression. Then difference expression of genes was analyzed by DESeq (1.30.0) with screened conditions as follows: expression difference multiple |log2FoldChange| > 1, significant P-value < 0.05. At the same time, We used R language Pheatmap(1.0.8) software package to perform bi-directional clustering analysis of all different genes of samples. We geted heatmap according to the expression level of the same gene in different samples and the expression patterns of different genes in the same sample with Euclidean method to calculate the distance and Complete Linkage method to cluster.

1. **GO and KEGG enrichment analysis**

we mapped all the genes to Terms in the Gene Ontology database and calculated the numbers of differentially enriched genes in each Term. Using topGO to perform GO enrichment analysis on the differential genes, calculate P-value by hypergeometric distribution method (the standard of significant enrichment is P-value <0.05), and find the GO term with significantly enriched differential genes to determine the main biological functions performed by differential genes. ClusterProfiler (3.4.4) software was used to carry out the enrichment analysis of the KEGG pathway of differential genes, focusing on the significant enrichment pathway with P-value.

1. **New transcript analysis**

On the basis of the existing reference genome, using the software StringTie (http://ccb.jhu.edu/software/stringtie/) to assemble the mapped reads, and comparing the splicing results with the known transcripts to obtain no annotations Transcripts of information.

1. **Differential variable shear analysis**

Using rMATS (3.2.5) software to analyze differential variable shear events. The main types of variable shear events analyzed are mainly SE, RI, MXE, A5SS, A3SS.

1. **SNP and indel analysis**

The Varscan program was used to obtain SNP and InDel sites, and the filtering criteria were: 1) SNP site base Q>20; 2) The number of Reads covering the site> 8; 3) The number of Reads supporting the mutation site> 2; 4) The p-value of SNP locus is <0.01. Analysis of variant sites.

1. **Transcription factor family analysis**

The prediction of transcription factors is to compare plants and animals with PlantTFDB (Plant Transcription Factor Database) and AnimalTFDB (Animal Transcription Factor DataBase) databases respectively to predict the transcription factor and the family information to which the transcription factor belongs.

1. **Exon difference analysis**

Using the DEXSeq package to analyze the difference in exon usage in RNA-seq experimental data, where the difference in exon usage refers to the relatively different exon usage due to experimental conditions.

1. **Interaction Analysis of Differential Gene Protein Network**

The STRING database (https://string-db.org/) is used for protein interaction analysis to reveal the relationship between target genes.
