# Supplementary material for: Extracellular Vesicles of Streptococcus anginosus Mediate Gastritis via Epithelial Barrier Disruption and Macrophage‐driven Inflammation
Source: Adv Sci (Weinh). 2026 Jan 30;13(19):e12494. doi: 10.1002/advs.202512494 (PMC13045418; doi:10.1002/advs.202512494)

Figure S1

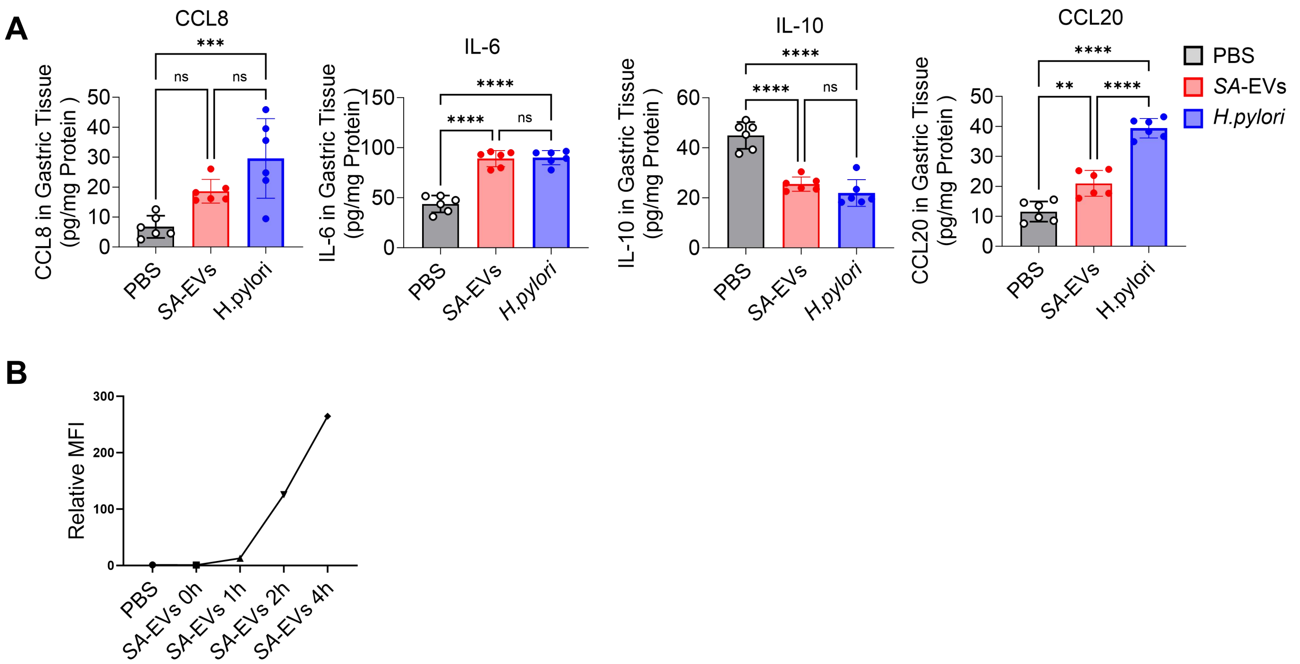
A
CCL8
CCL8 in Gastric Tissue (pg/mg Protein)
***
ns
ns
PBS
SA-EVs
H.pylori
IL-6
IL-6 in Gastric Tissue (pg/mg Protein)
****
****
ns
IL-10
IL-10 in Gastric Tissue (pg/mg Protein)
****
****
ns
CCL20
CCL20 in Gastric Tissue (pg/mg Protein)
****
**
****
PBS
SA-EVs
H.pylori
B
Relative MFI
PBS
SA-EVs 0h
SA-EVs 1h
SA-EVs 2h
SA-EVs 4h

Figure S2

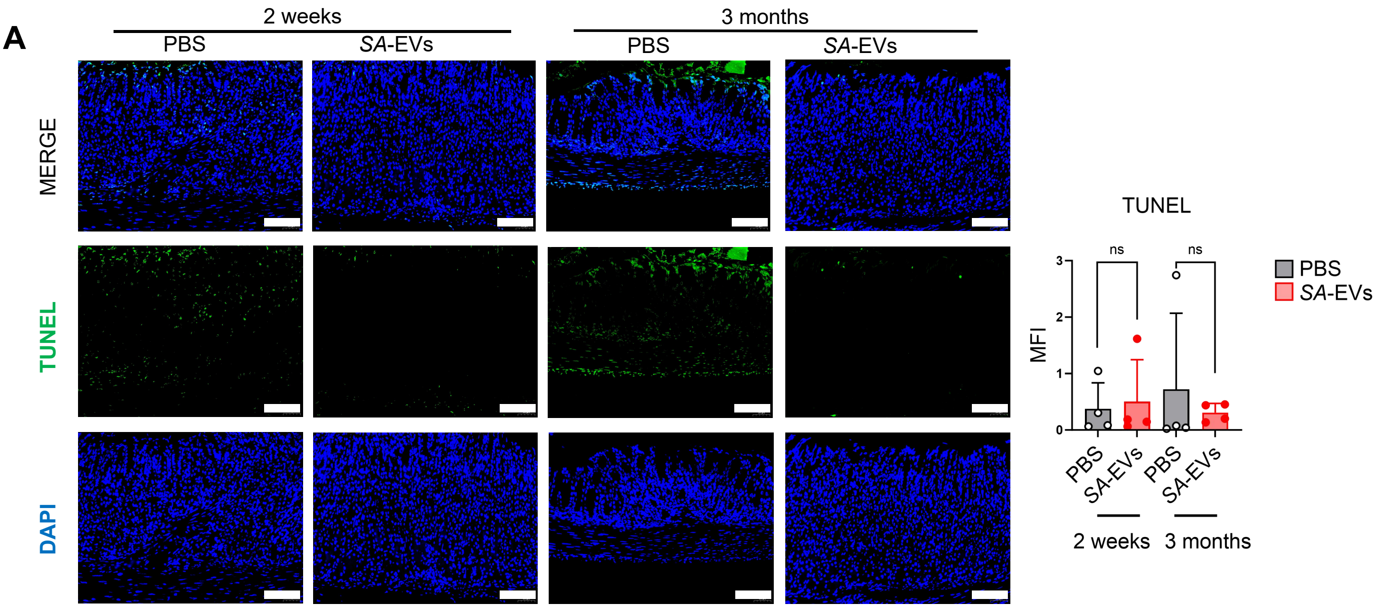
A
2 weeks
3 months
PBS
SA-EVs
PBS
SA-EVs
MERGE
TUNEL
DAPI
TUNEL
ns
ns
MFI
3
2
1
0
PBS
SA-EVs
PBS
SA-EVs
2 weeks
3 months
PBS
SA-EVs

Figure S3

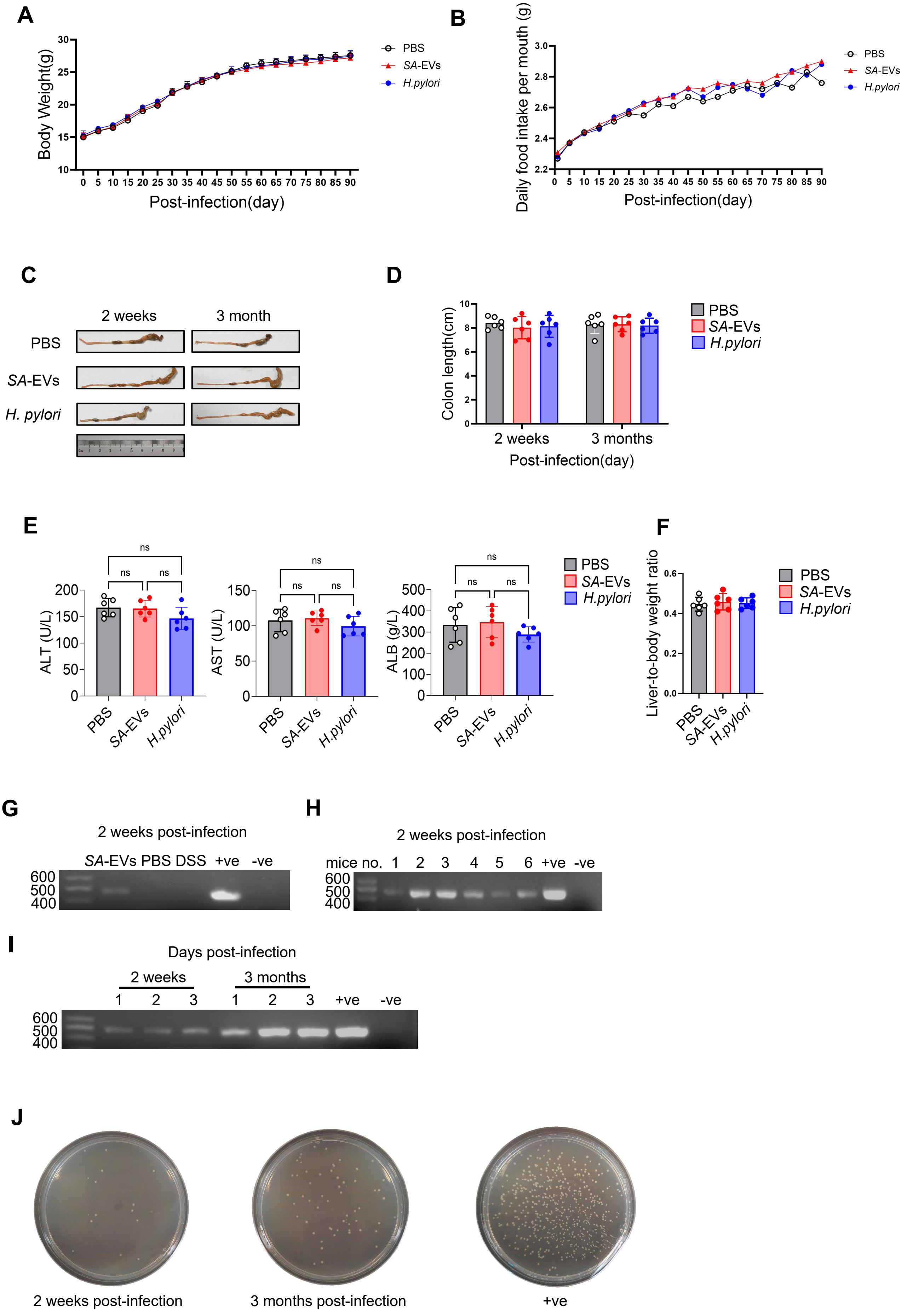

A
Body Weight(g)
Post-infection(day)
PBS
SA-EVs
H.pylori
B
Daily food intake per mouth (g)
Post-infection(day)
PBS
SA-EVs
H.pylori
C
2 weeks
3 month
PBS
SA-EVs
H. pylori
D
Colon length(cm)
2 weeks
3 months
Post-infection(day)
PBS
SA-EVs
H.pylori
E
ALT (U/L)
AST (U/L)
ALB (g/L)
ns
PBS
SA-EVs
H.pylori
F
Liver-to-body weight ratio
PBS
SA-EVs
H.pylori
G
2 weeks post-infection
SA-EVs PBS DSS +ve -ve
600
500
400
H
2 weeks post-infection
mice no. 1 2 3 4 5 6 +ve -ve
600
500
400
I
Days post-infection
2 weeks
3 months
1 2 3 1 2 3 +ve -ve
600
500
400
J
2 weeks post-infection
3 months post-infection
+ve

Figure S4

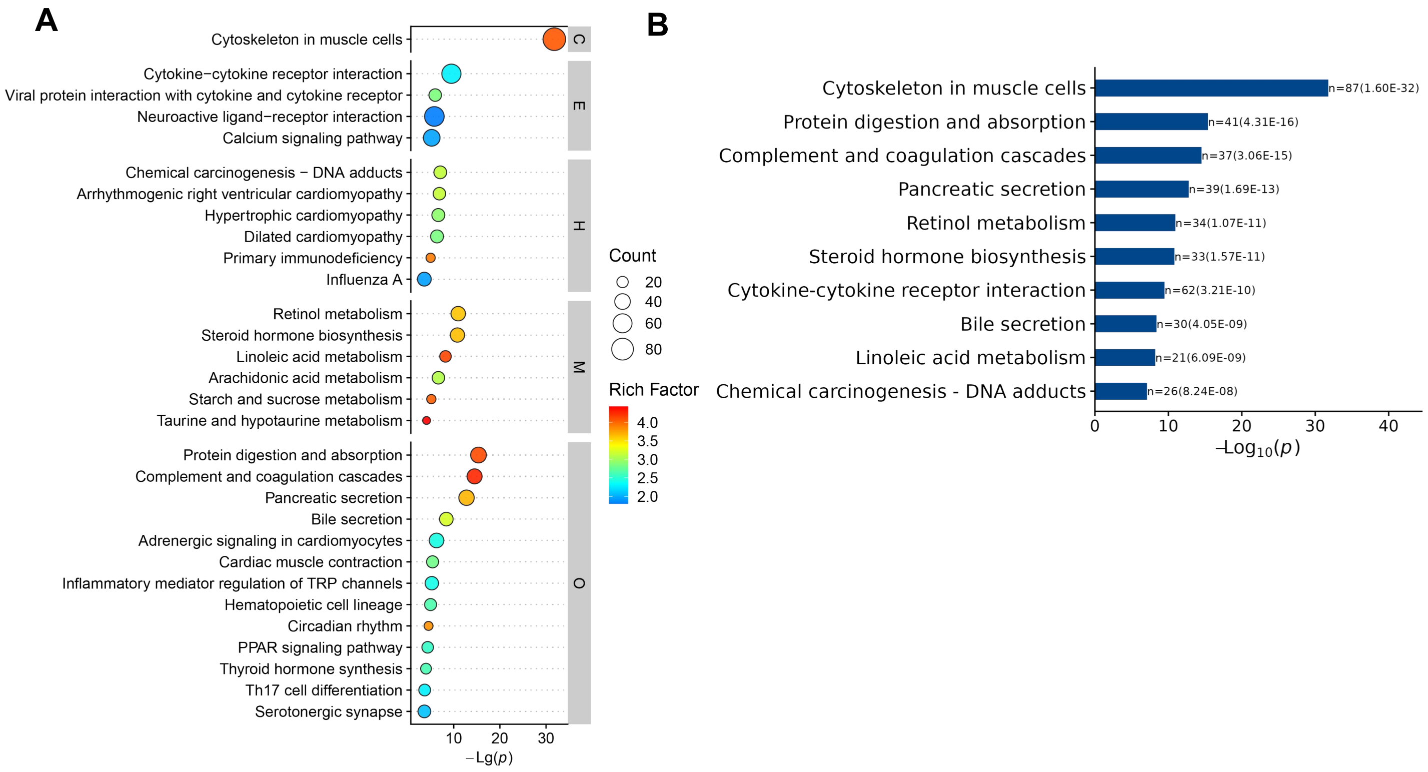
A
Cytoskeleton in muscle cells
Cytokine−cytokine receptor interaction
Viral protein interaction with cytokine and cytokine receptor
Neuroactive ligand−receptor interaction
Calcium signaling pathway
Chemical carcinogenesis − DNA adducts
Arrhythmogenic right ventricular cardiomyopathy
Hypertrophic cardiomyopathy
Dilated cardiomyopathy
Primary immunodeficiency
Influenza A
Retinol metabolism
Steroid hormone biosynthesis
Linoleic acid metabolism
Arachidonic acid metabolism
Starch and sucrose metabolism
Taurine and hypotaurine metabolism
Protein digestion and absorption
Complement and coagulation cascades
Pancreatic secretion
Bile secretion
Adrenergic signaling in cardiomyocytes
Cardiac muscle contraction
Inflammatory mediator regulation of TRP channels
Hematopoietic cell lineage
Circadian rhythm
PPAR signaling pathway
Thyroid hormone synthesis
Th17 cell differentiation
Serotonergic synapse
C
E
H
M
O
10
20
30
−Lg(p)
Count
20
40
60
80
Rich Factor
4.0
3.5
3.0
2.5
2.0
B
Cytoskeleton in muscle cells
Protein digestion and absorption
Complement and coagulation cascades
Pancreatic secretion
Retinol metabolism
Steroid hormone biosynthesis
Cytokine-cytokine receptor interaction
Bile secretion
Linoleic acid metabolism
Chemical carcinogenesis - DNA adducts
n=87(1.60E-32)
n=41(4.31E-16)
n=37(3.06E-15)
n=39(1.69E-13)
n=34(1.07E-11)
n=33(1.57E-11)
n=62(3.21E-10)
n=30(4.05E-09)
n=21(6.09E-09)
n=26(8.24E-08)
0
10
20
30
40
−Log10(p)

Figure S5

A

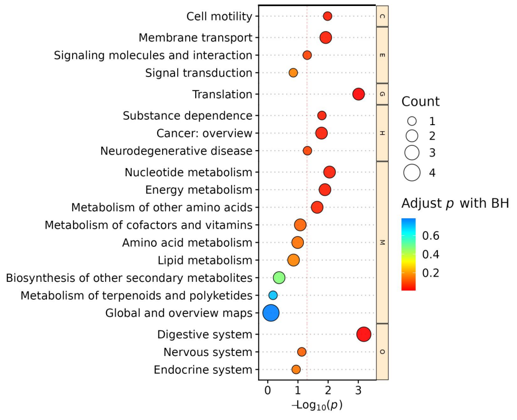

B

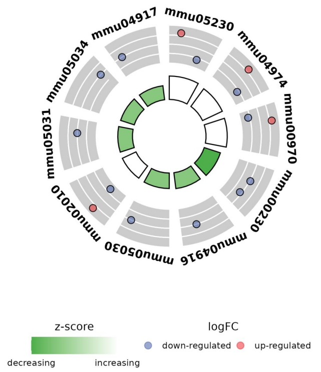

ID	Description
mmu05230	Central carbon metabolism in cancer
mmu04974	Protein digestion and absorption
mmu00970	Aminoacyl-tRNA biosynthesis
mmu00230	Purine metabolism
mmu04916	Melanogenesis
mmu05030	Cocaine addiction
mmu02010	ABC transporters
mmu05031	Amphetamine addiction
mmu05034	Alcoholism
mmu04917	Prolactin signaling pathway

C

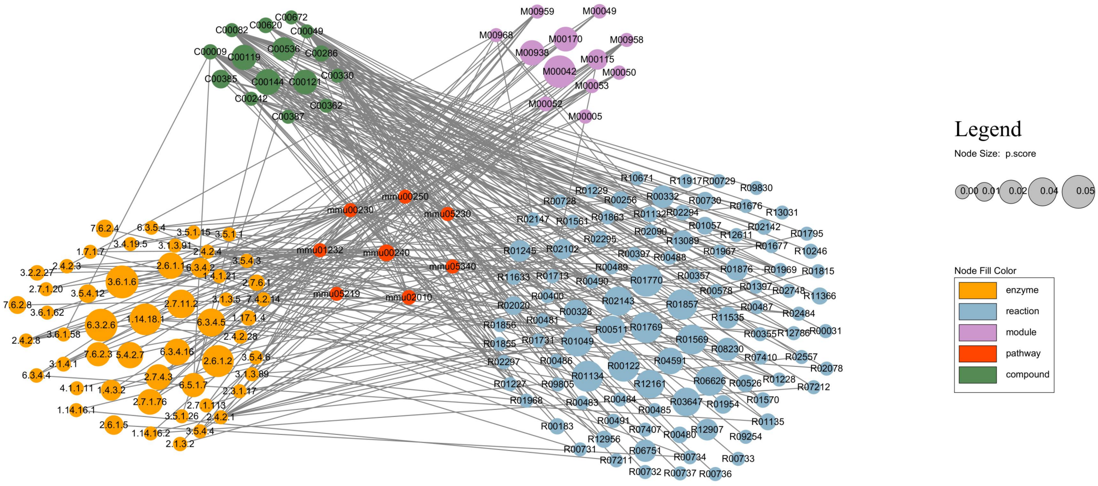

Figure S6

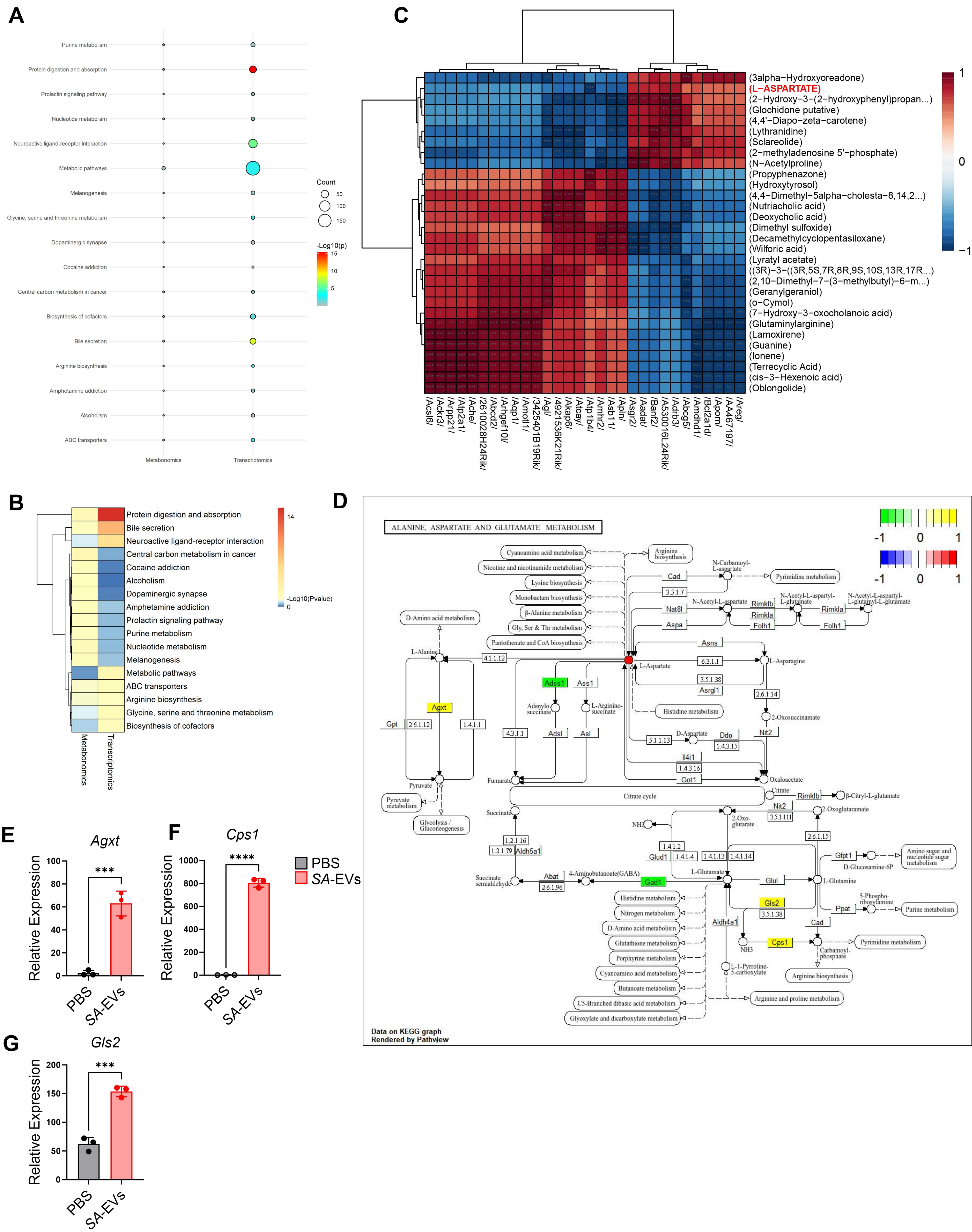
A
Purine metabolism
Protein digestion and absorption
Prolactin signaling pathway
Nucleotide metabolism
Neuroactive ligand-receptor interaction
Metabolic pathways
Melanogenesis
Glycine, serine and threonine metabolism
Dopaminergic synapse
Cocaine addiction
Central carbon metabolism in cancer
Biosynthesis of cofactors
Bile secretion
Arginine biosynthesis
Amphetamine addiction
Alcoholism
ABC transporters
Metabonomics
Transcriptomics
Count
50
100
150
-Log10(p)
15
10
5
C
(3alpha−Hydroxyoreadone)
(L−ASPARTATE)
(2−Hydroxy−3−(2−hydroxyphenyl)propan...)
(Glochidone putative)
(4,4'−Diapo−zeta−carotene)
(Lythranidine)
(Sclareolide)
(2−methyladenosine 5'−phosphate)
(N−Acetylproline)
(Propyphenazone)
(Hydroxytyrosol)
(4,4−Dimethyl−5alpha−cholesta−8,14,2...)
(Nutriacholic acid)
(Deoxycholic acid)
(Dimethyl sulfoxide)
(Decamethylcyclopentasiloxane)
(Wilforic acid)
(Lyratyl acetate)
((3R)−3−((3R,5S,7R,8R,9S,10S,13R,17R...)
(2,10−Dimethyl−7−(3−methylbutyl)−6−m...)
(Geranylgeraniol)
(o−Cymol)
(7−Hydroxy−3−oxocholanoic acid)
(Glutaminylarginine)
(Lamoxirene)
(Guanine)
(Ionene)
(Terrecyclic Acid)
(cis−3−Hexenoic acid)
(Oblongolide)
/Acsl6/ /Ackr3/ /Arpp21/ /Atp2a1/ /Ache/ /2610028H24Rik/ /Abcd2/ /Arhgef10l/ /Aqp1/ /Amotl1/ /3425401B19Rik/ /Agl/ /4921536K21Rik/ /Akap6/ /Atcay/ /Atp1b4/ /Amhr2/ /Asb11/ /Apln/ /Asgr2/ /Aadat/ /Banf2/ /A530016L24Rik/ /Adrb3/ /Abcg5/ /Amdhd1/ /Bcl2a1d/ /Apom/ /AA467197/ /Areg/
1
0
−1
B
Protein digestion and absorption
Bile secretion
Neuroactive ligand-receptor interaction
Central carbon metabolism in cancer
Cocaine addiction
Alcoholism
Dopaminergic synapse
Amphetamine addiction
Prolactin signaling pathway
Purine metabolism
Nucleotide metabolism
Melanogenesis
Metabolic pathways
ABC transporters
Arginine biosynthesis
Glycine, serine and threonine metabolism
Biosynthesis of cofactors
Metabonomics
Transcriptomics
14
-Log10(Pvalue)
0
D
ALANINE, ASPARTATE AND GLUTAMATE METABOLISM
-1 0 1
-1 0 1
Data on KEGG graph
Rendered by Pathview
E
Agxt
Relative Expression
***
PBS
SA-EVs
F
Cps1
****
G
Gls2
***
PBS
SA-EVs

Figure S7

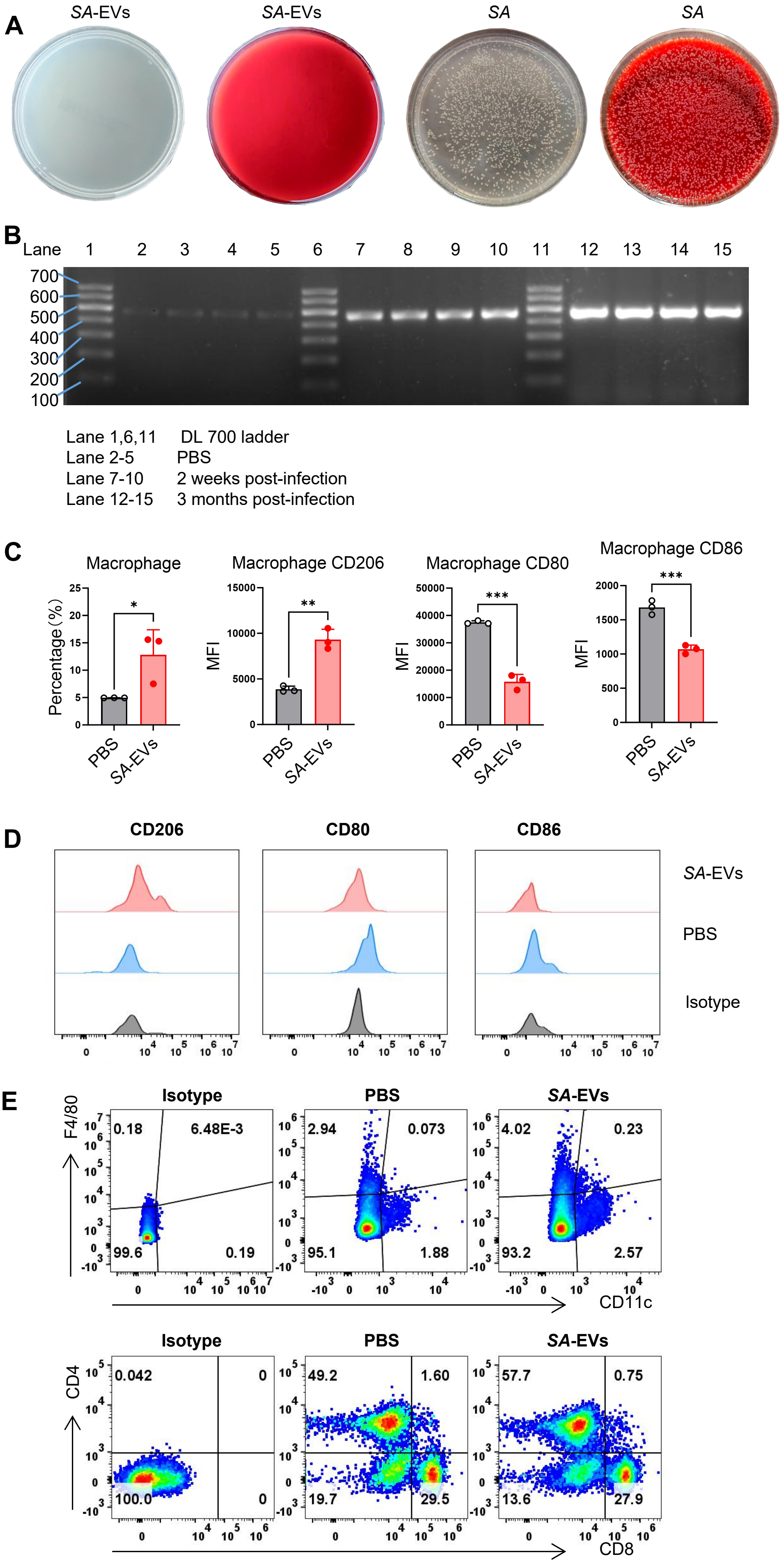
A
SA-EVs
SA-EVs
SA
SA
B
Lane 1 2 3 4 5 6 7 8 9 10 11 12 13 14 15
700
600
500
400
300
200
100
Lane 1,6,11 DL 700 ladder
Lane 2-5 PBS
Lane 7-10 2 weeks post-infection
Lane 12-15 3 months post-infection
C
Macrophage
Macrophage CD206
Macrophage CD80
Macrophage CD86
Percentage (%)
MFI
PBS
SA-EVs
*
**
***
D
CD206
CD80
CD86
SA-EVs
PBS
Isotype
E
Isotype
PBS
SA-EVs
F4/80
CD11c
0.18
6.48E-3
99.6
0.19
2.94
0.073
95.1
1.88
4.02
0.23
93.2
2.57
CD4
CD8
0.042
0
100.0
0
49.2
1.60
19.7
29.5
57.7
0.75
13.6
27.9

Figure S8

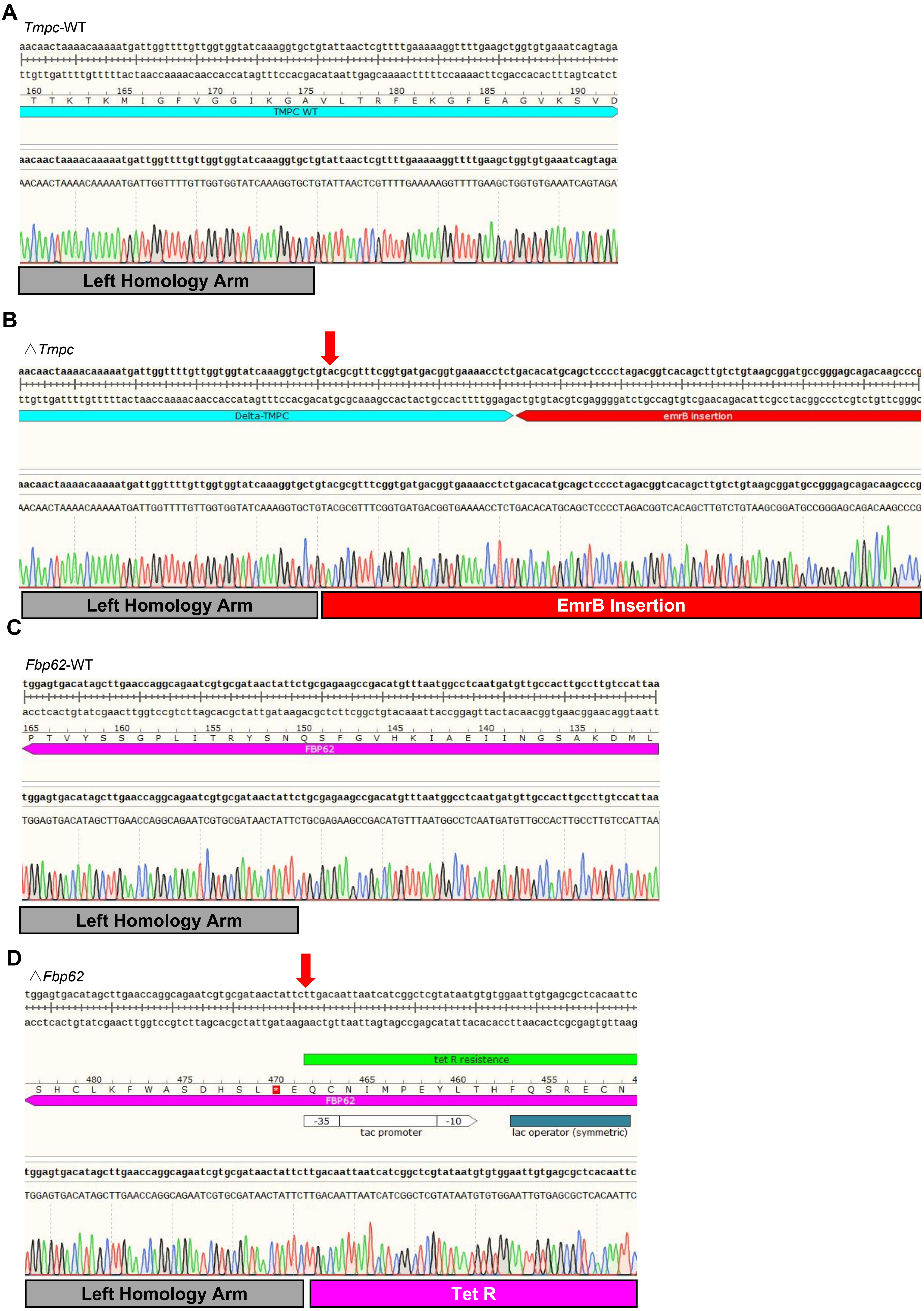
A
Tmpc-WT
TMPC WT
Left Homology Arm
B
△Tmpc
Delta-TMPC
emrB insertion
Left Homology Arm
EmrB Insertion
C
Fbp62-WT
FBP62
Left Homology Arm
D
△Fbp62
tet R resistence
FBP62
-35
-10
tac promoter
lac operator (symmetric)
Left Homology Arm
Tet R

Figure S9

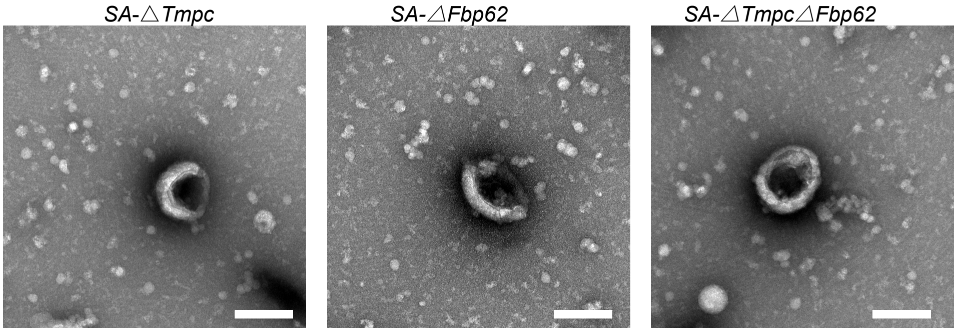

Supplement: Supplementary file 2 — Supporting File 2: advs74089‐sup‐0002‐Figure S1‐S9.pdf. [file ADVS-13-e12494-s008.pdf]
